# Expression of hemoglobin-α and β subunits in human vaginal epithelial cells and their functional significance

**DOI:** 10.1371/journal.pone.0171084

**Published:** 2017-02-08

**Authors:** Debarchana Saha, Swanand Koli, Mandar Patgaonkar, Kudumula Venkata Rami Reddy

**Affiliations:** Division of Molecular Immunology and Microbiology (MIM), National Institute for Research in Reproductive Health (NIRRH), Parel, Mumbai, India; University of Hyderabad, INDIA

## Abstract

Hemoglobin (Hb) is a major protein involved in transport of oxygen (O_2_). It consists of Hb-α and Hb-β subunits, which are normally expressed by cells of erythroid lineage. However, till recently, it was not known whether non-erythroid cells like vaginal cells synthesize Hb and whether it has any functional significance. Therefore, we designed the following objectives: (1) to establish *in-vitro* culture system of human primary vaginal epithelial cells (hPVECs), (2) to determine whether Hb-α and Hb-β proteins are truly synthesized by hPVECs, (3) to evaluate the effect of LPS (lipopolysaccharide) on the expression of Hb-α and Hb-β proteins (4) to decipher the significance of the Hb-α and Hb-β expression in hPVECs and (5) to determine the molecular mechanism regulating the expression of Hb-α in hPVECs. To accomplish these studies, we applied a battery of assays such as RT-PCR, qRT-PCR, Flow cytometry, western blot, and immunofluorescence, Electrophoretic mobility shift assay (EMSA) and chromatin immunoprecipitation (ChIP). The results revealed the expression of Hb-α and Hb-β at both mRNA and protein level in hPVECs. The expression was significantly upregulated following LPS treatment (10μg/ml for 6 hrs) and these results are comparable with the expression induced by LPS in human vaginal epithelial cell line (VK2/E6E7). These cells constitutively produced low levels of pro-inflammatory (IL-6) and anti-inflammatory (IL-10) cytokines. Also, the response of phosphorylated (p65)-NF-*κ*B to LPS was upregulated with increased expression of IL-6, Toll-like receptor-4 (TLR4) and human beta defensin-1 (hBD-1) in hPVECs and VK2/E6E7 cells. However, Bay 11–7082 treatment (5μM for 24 hrs) could neutralize the effect of LPS-induced p65-NF-κB activity and represses the production`of Hb-α and Hb-β. The results of EMSA revealed the presence of putative binding sites of NF-κB in the human Hb-α promoter region (nt-115 to -106). ChIP analysis confirmed the binding of NF-*κ*B to Hb-α promoter. In conclusion, the present findings revealed for the first time that hPVECs synthesized Hb-α and Hb-β and the expression is comparable with the expression of VK2/E6E7 cells. The identification of NF-*κ*B regulatory sequences in Hb-α promoter, whose activation is associated with immune response of hPVECs, indicating Hb-α and Hb-β may act as an endogenous antimicrobial defense protein against vaginal inflammation/infections.

## Introduction

Vaginal epithelium of the female reproductive tract (FRT) is constantly exposed to a large number of pathogens, which cause sexually transmitted infections (STIs) [1, 2]. Vaginal epithelial cells (VECs) of FRT play an important role in initiation, regulation and resolution of both innate and adaptive immune functions [3, 4]. As part of innate immunity, VECs synthesize and secrete antimicrobial peptides (AMPs) such as β-defensins (BDs), neutrophil peptides 1–3 (NP-1-3), secretory leukocyte protease inhibitor (SLP1), lysozyme, lactoferrin etc, into vaginal lumen [5]. Several studies have indicated that hemoglobin (Hb) and peptides derived from it (e.g. hemocidins) play a critical role in the protection of vaginal epithelium by acting against the invading pathogens [2, 6, 7].

Hb belongs to an ancient family of heme-associated, iron-containing oxygen-transport metallo-protein in the red blood cells (RBCs) [8]. Hb is involved in the transport of CO_2_ (~20–25% of the total). The major Hb form in adults is a tetrameric metalloprotein (~64500 Da) made up of two α- and two β-polypeptide chains (α2β2), which are encoded by two genes located on chromosomes 16 and 11 respectively [8]. The Hb-α gene is embedded in an open chromatin conformation, whereas β-globin gene cluster is packaged into an inactive heterochromatin in all the cell types [9]. However, it is not known why Hb-α and Hb-β genes are regulated so differently in vaginal epithelial cells even though when their expression is always tightly linked.

It has been documented that Hb is expressed only by the cells of erythroid lineages. However, this old belief has been challenged by many investigators who have proved that Hb expression is much more versatile than previously assumed [10, 11]. Recent advances in Hb research have revealed its expression in a wide variety of non-erythroid cells ranging from mouse macrophages [11], lungs [12], retinal pigment epithelial cells [13], mesangial cells of rat and human [14], hepatocytes [15], neuronal/glial cells [16], kidney cells [17], endometrial cells [18], lens of the eye [19], cervical cell lines (SiHa and CaSki) [20], alveolar epithelial cells of rat, humans [21–23] and injured sciatic nerves [24]. However, the role of Hb in these cells is poorly understood. In glial cells it has been shown that Hb functions as an antioxidant and regulate iron metabolism [15]. Several authors have also reported the presence of Hb in uterine fluid [18], placental fluid [25] and menstrual fluid in human [26, 27]. In our recent study, Saha et al [7] demonstrated the expression of Hb-α and Hb-β in human vaginal epithelial cell line, VK2/E6E7. However, in this study we could not authenticate the expression of Hb-α and Hb-β since this cell line was immortalized with human Papillomaviral gene (16/E6E7) [28]. To rule out this possibility, currently we lacked established standard in *vitro* culture system to obtain pure population of human primary vaginal epithelial cells (hPVECs). Moreover, it is not known clearly why hPVECs synthesize Hb-α and Hb-β and the mechanism involved in this process. In the current study, we sought to: 1) establish *in-vitro* culture system of hPVECs, 2) investigate whether Hb-α and Hb-β proteins are synthesized by hPVECs, 3) assess whether the expression is modulated by gram-negative bacterial membrane protein, lipopolysaccharide (LPS), 4) evaluate the biological significance of Hb-α and Hb-β expression in hPVECs and 5) determine the pathway/s mediating the expression of Hb-α in hPVECs. Our results demonstrated that Hb-α and Hb-β proteins were constitutively expressed by hPVECs and are comparable with the expression observed in VK2/E6E7 cell line. The expression of interleukin-6 (IL-6), human beta defensin-1(hBD-1), toll-like receptor-4 (TLR-4) and phosphorylated (p65)-NF-*κ*b(p65-NF-κB) was upregulated by LPS and attenuated by NF-κB inhibitor, Bay 11–7082 through the activation of TLR4-NF-κB downstream pathway. The ALGGEN—PROMO online software analysis indicated the presence of NF-κB binding site on Hb-α promoter. EMSA and ChIP results confirmed p65-NF-κB enhances the transcription of Hb-α by binding to Hb-α promoter region (nt-115 to -106).

## Materials and methods

### Procurement human vaginal epithelial cell line (VK2/E6E7) and culture

Vaginal epithelial cell line (VK2/E6E7) is a well differentiated human vaginal epithelial cells derived from normal vaginal mucosa of a healthy premenopausal women undergoing vaginal repair surgery. The cell line was developed by immortalizing with human papillomavirus, 16/E6E7 by Dr. Raina Fichorova, Maghee Women’s Research Institute, Harvard Medical School, Boston, USA. This cell line was obtained as a kind gift from Dr. Fichorova. Mycoplasma free VK2/E6E7 cells were maintained in keratinocyte serum-free medium (KSFM) supplemented with bovine pituitary extract (BPE, 50 μg/ml), recombinant epidermal growth factor (EGF, 0.1 ng/ml), 0.4 mM CaCl_2_ and 1X antibiotic cocktail consisting of Penicillin, Streptomycin and Amphotericin (Sigma-Aldrich, USA). Cell line was maintained at 37°C in a humidified atmosphere containing 5% CO_2_. The morphological and immunological characteristics of these cells closely resembled vaginal epithelial tissue of origin. This cell line maintained a stable phenotype after more than 1 year of continuous passage and expressed characteristic marker of epithelial cells, cytokeratin-13 (CK13). This cell line is being used by many investigators as *in vitro* model for various studies including vaginal infection, testing of microbicide compounds for cell viability and toxicity [28] etc. In the present study, this cell line was used as positive control to compare the expression of Hb-α and Hb-β proteins with that of human primary vaginal epithelial cells (hPVECs). In this study, we referred this cell line as VK2/E6E7 cells.

### Isolation of primary human vaginal epithelial cells (hPVECs)

#### Vaginal tissue collection

The use of vaginal tissue samples has been approved by the NIRRH Ethics Committee for Clinical studies (D/ICEC/Sci-79/130/2013) and Institutional Ethics Committee of the collaborating hospital, Seth G. S. Medical College & KEM hospital (EC/GOVT-9/2013). Written informed consents were obtained from all subjects before inclusion in the study. Vaginal tissue samples (n = 15) from women (aged between 18–40 yrs) undergoing surgery for vaginal prolapse were collected in physiological saline during the period from June 2014 to March 2016. The samples were processed within 30 min after the collection.

#### Culture of hPVECs

The hPVECs culture was set-up with 1 cm^2^ of vaginal tissue fragments using 0.1% trypsin/ 0.01% EDTA. Following enzymatic dissociation, hPVECs were passed through a sterile 70 μm pore-size nylon membrane. The filtrate was pelleted by centrifugation at 1500 rpm for 5 mins. The supernatant was discarded, vaginal epithelial cell-enriched pellet was suspended in KSFM. The cells were seeded onto collagen IV (10 mg/mL) coated flasks and maintained in serum-free and estrogen-free conditions. The cell viability was determined in triplicate wells by trypan blue exclusion assay using a hemocytometer. The unstained viable cells were counted on the basis of their trypan blue exclusion, while dead cells were stained blue. Cells were cultured in KSFM after supplemented with bovine pituitary extract (BPE) and epidermal growth factor (EGF) as described above for VK2/E6E7 cells. The morphology of cells was evaluated initially by phase contrast microscopy. The purity of the hPVECs enriched pellet (>98%) was determined by enumerating five separate fields of 100 cells and is expressed as the mean percentage of hPVECs with an epithelial morphology. Purity was further checked by immunofluorescence and flow cytometry assays using vimentin (stromal cell marker) and cytokeratin-13 (epithelial stratification marker).

### Isolation of RBCs

Vaginal tissue along with adherent blood were washed thrice in 1 ml of physiological saline. After the separation of tissue, samples were centrifuged at 1500 rpm for 5 mins. Supernatants were discarded, RBCs pellet was collected, washed twice with saline and used as positive control to check for possible contamination of hPVEC cultures with RBCs using RBC specific marker, solute carrier 4A protein-1 (*SLC4A1*) [29] by immunofluorescence and western blot methods.

### Stimulation of hPVECs and VK2/E6E7 cells with LPS

Cells were detached from 75-cm^2^ culture flasks using trypsin. They were washed twice in culture medium, counted, seeded into 12well tissue culture plates (Nunc-Nalgene, USA) at 2.5 X10^5^ cells/well and incubated in a 5% CO_2_ atmosphere at 37°C. Our previous studies showed that VK2/E6E7 cells responds to LPS when stimulated with a non-toxic concentration (10 μg/ml) [30]. Therefore, this concentration was selected as an optimum dose for all the experiments. Briefly, both hPVECs or VK2/E6E7 cells were grown in KSFM in a 12 well culture plates and divided into four groups: untreated group -1 (hPVECs) and group-2 (VK2/E6E7) cells (2.5 x 10^5^ cells/well) cultured in KSFM, and group-3 (hPVECs) and group-4 (VK2/E6E7) cells treated with LPS (10 μg/ml) for 6 hrs. At the end of the treatments, the culture supernatants were collected and stored at -80°C. The cells were harvested and washed twice in cold PBS. A portion of the hPVECs or VK2/E6E7 cells were used for immunofluorescence experiments and to prepare nuclear extracts for EMSA and ChIP assays. Remaining cells were used to extract total mRNA (for RT-PCR & qRT-PCR) and proteins (for western blot).

### Indirect immunofluorescence (IIF) analysis

To check the epithelial cell purity, we used mouse monoclonal cytokeratin-13 antibody (CK-13) (C0791, Sigma) and rabbit monoclonal vimentin antibody (ab92547, abcam) (1:100 dilutions in PBS). Primary antibody signals were detected using respective FITC-conjugated goat anti-mouse and anti-rabbit secondary antibodies (1:200 dilutions in PBS), (Invitrogen, USA). Cells treated only with secondary antibody were used as negative control. After washing thrice with PBS, cover slips were mounted on glass slides using Vector shield anti-fading reagent and observed under the confocal microscope attached to a Zeiss Axiovert 100 microscope, using a 63 X Plan-Apochromatic (Nomarski Differential Interference Contrast optics) under oil immersion objective.

To localize solute carrier 4A protein-1 (SLC4A1) (in RBCs, hPVECs and VK2/E6E7 cells) and p65-NF-κB (in hPVECs and VK2/E6E7 cells), we used rabbit primary antibody (ab108414, abcam) and goat anti-rabbit FITC conjugated secondary antibody (Invitrogen, USA) respectively. Rest of the protocol followed was the same as described above.

The expression of Hb-α and Hb-β in hPVECs and VK2/E6E7 cells was done by indirect immunofluorescence (IIF) analysis. For this, hPVECs and VK2/E6E7 cells were grown on cover slips as described previously [31]. Briefly, cells were fixed in 4% para-formaldehyde for 10 min. After the fixation cover slips were washed with PBS. The cells were blocked with 5% BSA in PBS for 1hr at RT and then treated overnight at 4°C with rabbit anti-Hb-α (ab92492, abcam) and anti-Hb-β (ab172019, abcam) antibodies (1:100 dilutions in PBS). After incubation, cells were washed and treated with FITC labeled goat-anti rabbit secondary antibody (Invitrogen, USA) (1:200 dilution in PBS) for 1 hr at room temperature (RT) in dark. The cells were counter stained with DAPI.

### Flow cytometry analysis

For flow cytometric analysis of intracellular localization of cytokeratin-13 (CK-13) and vimentin, hPVECs and VK2/E6E7 cells growing in culture were washed and treated with fixation buffer (4% paraformaldehyde) followed by permeabilization buffer (0.1% saponin). The cells were incubated for 1 hr at RT with rabbit monoclonal CK-13 or rabbit monoclonal vimentin antibody (1:1000) diluted in PBS with 1% FBS. After incubation, cells were washed thrice with PBS and then stained with FITC labeled goat anti-rabbit secondary antibody. After incubation for 1 hr at RT, cells were washed thrice and re-suspended in PBS in 1% FBS, and analyzed by flow cytometer (Becton Dickinson, USA) available at the central equipment facility of NIRRH, Mumbai. The percentage positive cells and mean fluorescence were calculated. Data for 10,000 cells were collected for each sample. Secondary antibody alone was included as negative controls.

### Western blot analysis

The immunofluorescence results were further checked by western blot analysis using specific antibodies against Hb-α and Hb-β proteins. The hPVECs and VK2/E6E7 cells were lysed with ice cold RIPA buffer supplemented with a protease and phosphatase inhibitor cocktail. Cell lysates were then centrifuged at 10,000 rpm for 20 minutes at 4°C and supernatants were collected. The protein concentration was determined by the BCA assay (Pierce, USA). Protein (30 μg/well) from the extract of hPVECs and VK2/E6E7 cells was loaded on 10% SDS-PAGE and proteins were separated at 110V for 2 hrs. Gel was transferred to polyvinylidene difluoride (PVDF) membranes (BioRad, USA). Ponceau-S stain was used to evaluate the transfer efficiency. The strips were blocked with 5% Nonfat dry milk (NFDM) powder in 0.1% TBST for 1 hr at RT and strips were then probed with anti-Hb-α and anti-Hb-β antibody. The membranes were washed with 0.1% TBST buffer for 1 hr followed by probing with horseradish peroxidase (HRP)-conjugated goat anti-rabbit secondary antibody (1:1000 dilution in PBS) (Sigma-Aldrich, USA). The membranes were then washed 0.1% TBST for 1 hr and bound antibody was detected by enhanced chemi-luminescence (ECL) detection reagents (GE Biosciences, USA) according to manufacturer’s instructions. Relative expression was calculated and normalized to the values of β-actin (ab8224, abcam) control. Intensities of the bands were quantified using Quantity-one software by densitometry scanning (Bio-Rad, USA). Same protocol was followed for the determination of SLC4A1 expression in RBCs, hPVECs and VK2/E6E7 cells.

### RNA extraction and reverse transcription (RT)-PCR analysis

Total RNA was extracted from hPVECs or VK2/E6E7 cells (seeded at a density of 1x10^6^/ well in 6-well plates) of untreated, TLR4 antibody (sc-13593 FITC, Santa Cruz) treated (2μg/ml, for 1 hr), LPS-induced (10 μg/ml, for 6 hrs) and Bay 11–7082 treated (5 μM, for 24 hrs) before or after inducing with LPS (10 μg/ml, for 6 hrs) using Trizol reagent according to the manufacturer’s protocol (Invitrogen, USA). RNA samples were quantified using a NanoDrop ND-1000 spectrophotometer. RNA was converted to cDNA by single strand cDNA synthesis kit (Bio-Rad, USA). The cDNAs of respective samples were used to amplify *Hb-α*, *Hb*-*β*, human β defensin-1 **(***hBD-1*), *TLR4* and housekeeping gene *Gapdh* using gene specific primers. The primer sequences and annealing temperatures identified for each gene are given in Table 1. The PCR reaction included an initial activation step at 95°C for 2 min, followed by 35 cycles at 94°C for 30 secs, annealing at 60°C for 30 secs, extension at 72°C for 30 sec and a final extension at 72°C for 7 min. *Gapdh* served as internal loading control. PCR products were visualized by electrophoresis on a 2.0% agarose gel. The gels were scanned by using a Gel Documentation System (Gel Doc 2000, Bio-Rad, USA) and intensity of the bands was quantified by “Quantity-One” software. The products obtained were eluted from the gel and sequenced using an ABI PRISM 377 DNA sequencer (Applied Biosystems, USA) at NIRRH central sequencing facility.

**Table 1:** List of primer sequences used for RT-PCR and qRT-PCR analysis in the study.

Gene	Sequence	Accession No.	Product size (bp)	Annealing Temp (°C)
*Hb-α*	F-5’-ATGGTGCTGTCTCCTGCCGACAAG-3’	NM_000558.4	429	62
	R-5’-TTAACGGTATTTGGAGGTCAGCACGG-3’	-	-	-
*Hb-β*	F-5’- ATGGTGCATCTGACTCCTGAGG-3’	NM_000518.4	444	62
	R-5’-TTAGTGATACTTGTGGGCCAG-3’	-	-	-
*TLR4*	F-5-′ CCGCTTTCACTTCCTCTCAC-3′	NM_138554.4	182	58
	R- 5′- CATCCTGGCATCATCCTCAC-3’		-	-
*hBD1*	F-5′- TGTCAGCTCAGCCTCCAAAG-3’	NM_005218.3	139	60
	R- 5′- TACCACCTGAGGCCATCTCA-3’	-	-	-
*Gapdh*	F-5-′GAGTCAACGGATTTGGTC-3′	NM_002046.5	238	58
	R-5-′TTGATTTTGGAGGGATCTC-3’		-	-

### Quantitative PCR (qPCR) analysis

Total RNA was extracted from untreated, LPS treated and Bay 11–7082 treated hPVECs and VK2/E6E7 cells using TRIzol reagent. Total RNA (1 μg) was reverse transcribed into the first-strand cDNA using iScript cDNA synthesis kit (Bio-Rad, USA) according to the manufacturer’s instructions. The oligonucleotide specific primers for *Hb-α*, *Hb-β* and *Gapdh* were the same used for RT-PCR. For each primer pair, reaction efficiency was estimated by the amplification of serial dilution of vaginal cell cDNA pool over a 10-fold range. The expression of *Hb-α* and *Hb-β* transcripts were analyzed with respect to *Gapdh* (housekeeping gene) by CFX96 real-time PCR system using SYBR Green chemistry (Bio-Rad,USA). The amplification conditions were the same as mentioned for RT-PCR. The fluorescence emitted at each cycle was collected after the extension step of each cycle. The homogeneity of the PCR amplicons was verified by studying the melt curve. Mean Ct values generated in each experiment using the CFX Manager software (Bio-Rad, USA) were used as an indicator to obtain fold change in the expression of target genes normalized to *Gapdh* mRNA. The relative expression levels in terms of fold change were calculated by 2-ΔΔCt method [32].

### Quantification of cytokines by ELISA

The levels of pro-inflammatory (IL-6) and anti-inflammatory/Th2-immunoregulatory (IL-10) cytokines were measured in the culture supernatants of hPVECs and VK2/E6E7 cells by enzyme-linked immunosorbent assay (ELISA) using commercially available human quantikine ELISA kits (D6050 for IL-6) and (D1000B for IL-10) (R&D Systems, Minneapolis, USA) as described earlier [33]. These cytokines were chosen based on the fact that they represent important members of innate immune responses of human vaginal mucosal epithelium. Spent media was collected from unstimulated and LPS stimulated (10μg /ml) hPVECs and VK2/E6E7 cells at 6, 12 and 24 hrs post treatment. Supernatants were filtered through 0.22μm-pore-size filters. A 50 μl volume of undiluted supernatant was used to measure these cytokines in triplicate samples. Standard curves were generated by respective recombinant human cytokines as previously described [34]. The absorbance values and concentrations of each cytokine were determined by using automated microplate ELISA reader at 492 nm (Bio-Tek Corp, Winooski, Germany). The sensitivity of each set of antibodies ranged from 4 to 15 pg/ml. LPS interference with cytokine detection was ruled out by spiking known amounts of recombinant IL-6 and IL-10 by measuring the percent cytokine recovery from LPS supplemented medium versus the plain medium and the values expressed as picograms of cytokine/ ml.

### Treatment of hPVECs and VK2/E6E7 cells with NF-κB inhibitor, Bay 11–7082

To determine whether phosphorylated p65-NF-κB has any role on the expression of Hb-α and Hb-β in hPVECs and VK2/E6E7 cells, p65- NF-κB activity was inhibited using a chemical compound, Bay 11–7082 (Sigma-Aldrich, USA). This compound is known to inhibit p65-NF-κB binding to DNA by preventing phosphorylation of the Inhibitor of κB (IκB) by the IκB Kinase (IKK) [35]. For initial, standardization studies, VK2/E6E7 cells were treated with 2.5 and 5 μM concentrations of Bay 11–7082 for 6, 12, 24 or 48 hrs to find out an appropriate dose and duration of treatment at which maximum inhibition of p65-NF-κB. Based on the preliminary results, 5 μM concentrations of Bay 11–7082 and 24 hrs duration were selected and accordingly hPVECs and VK2/E6E7 cells have been treated. We also included an additional group wherein cells were stimulated with LPS (10 μg/ml for 6 hrs), after washing they were treated with Bay 11–7082 (5 μM for 24 hrs). The expression of Hb-α and Hb-β was determined by RT-PCR. The cells treated with DMSO were used as vehicle control.

### Determination of p65-NF-κB by ELISA and immunofluorescence assays

To determine p65-NF-κB by ELISA, transcription Factor Assay Kit was used (ab133112, abcam). After the treatment of cells with LPS (10 μg/ml for 6 hrs), they were lysed with hypotonic HEPES lysis buffer (pH 7.4) and centrifuged at 1000 g for 10 min at 4°C, supernatants were collected and used for the determination of intracellular p65- NF-κB by ELISA as described earlier [30]. Briefly, a specific double stranded DNA (dsDNA) sequence containing the p65-NF-κB response element was immobilized onto the bottom of wells of a 96-well plate. p65-NF-κB contained in a nuclear extract, binds specifically to the p65-NF-κB response elements. p65-NF-κB was detected by addition of specific primary antibody directed against p65-NF-κB. A secondary antibody conjugated to HRP was added and the absorbance was read at 450 nm using spectrophotometer.

### Computational analysis of NF-κB binding site in Hb-α promoter

Sequences of mammalian genomic regions upstream of Hb-α translation start site (TSS) were retrieved from the NCBI genome sequence database. Exact matches to NF-κB consensus-binding site (GGGRNNYYCC) or its reverse complement (GGRRNNYCCC), as well as sequences with a conservative one-nucleotide deviation (R—R or Y—Y) were analysed and identified using the online software ALGGEN-PROMO–(http://alggen.lsi.upc.es/cgibin/promo_v3/promo/promoinit.cgi?dirDB=TF-8.3)

### Preparation of cell lysates and Electrophoretic mobility shift assay (EMSA)

Nuclear extracts were prepared as described previously [36]. Commercially synthesized oligonucleotide probes corresponding to Hb-α promoter (Sigma-Aldrich, USA) were used to detect the interactions of NF-κB and Hb-α promoter. A 20 bp probe covering the Hb-α promoter sequences between nt-115 to -106 was used for EMSA. The sequences of NF-κB wild-type probe (F-5′-CCGCCCGGGACTCCCCTGCG-3′; R-5’- CGCAGGGGAGTCCCGGGCGG-3’) and the sequences of mutant-type probe (F-5′-CCGCCCGGGCCTGACCTGCG-3′; R-5’-CGCAGGTCAGGCCCGGGCGG-3’) and NF-κB standard probe (F-5'-AGTTGAGGGGACTTTCCCAGGC-3'; R-5'–GCCTGGGAAAGTCCCCTCAACT-3’) are given in the parentheses. Heat-annealed duplex oligonucleotide probes were DIG-11-ddUTP labeled using recombinant TdT as per the manufacturer’s protocol (Roche Diagnostics, Indianapolis, IN, USA).

Nuclear extracts from untreated or LPS-treated hPVECs cells were prepared using a nuclear protein extract Kit as per the manufacturer’s protocol (Sigma-Aldrich St. Louis, MO, USA). Protein concentrations were determined using a BCA Protein Assay Kit (Pierce, Rockford, USA). For the super-shift binding assay, antibody against p65-NF-kB (Cell Signaling Technologies Inc., USA) was incubated with nuclear extract before a labeled probe was added. The binding reaction was performed by competition experiments, unlabeled double-stranded wild-type probes at final concentrations of 4 (50X), 8 (100X) and 16 (200X) pmol were added simultaneously with 0.08 pmol of DIG-labeled NF-κB probe (or unlabeled DNA for competitive assay) to the binding reaction with a total 1 μg of nuclear protein.

### Chromatin immunoprecipitation (ChIP) assay

To further confirm the *in vitro* binding of p65-NF-κB on Hb-α promoter, ChIP assay was performed using the Simple ChIP plus Enzymatic Chromatin IP Kit as per the manufacturer’s protocol (Cell Signaling Technology, USA). Briefly, untreated and LPS stimulated hPVECs (1 x10^6^ cells) were sonicated (15 cycles for 60 seconds on, 60 seconds off) at 100% power to shear DNA to achieve fragments that were 200–1000 bp in size. After that the samples were centrifuged and the supernatant containing the sheared chromatin was collected. The sonicated chromatin was incubated with antibodies against NF-*κ*B (Cell Signaling Technology, USA) or normal rabbit serum (IgG) (Santa Cruz, USA) overnight at 4°C on a rotor. Ten percent of the total genomic DNA from nuclear extracts was used as the input. Purified immune-precipitated DNA and input DNA were recovered by phenol/chloroform extraction and ethanol precipitation and used as templates for PCR with the following primers to amplify NF-*κ*B binding site on human Hb-α promoter region (F-5’-GGGACTCCCCTGCGGTCCA -3’ and R-5’-CGAGCGCGCCAGGGTTTATG -3’) were designed. A total of 10% of the chromatin DNA used for immunoprecipitation was similarly subjected to PCR analysis and indicated as ‘input’. The number of PCR cycles was: 36 for all the ChIP experiments and 26 for the input samples. A 106 bp PCR product was visualized on 2% agarose gel.

### Statistical analysis

Except where indicated, representative results from at least three experiments are presented in the figures. Data are expressed as means, and error bars represent as the mean ± standard deviation (SD). The statistical significance of differences between two means was evaluated using the two-tailed unpaired Student’s t-test. Values of P< 0.05 were considered to be statistically significant. The data were analyzed by GraphPad Prism 5 (GraphPad Software, CA, USA). The following notations have been used to denote p values in the figures: *: P < 0.05; **: P < 0.01; ***: P <0.001.

## Results

### Characterization of human primary vaginal epithelial cells (hPVECs)

#### hPVECs express cytokeratin-13 but not vimentin (Immunofluorescence analysis)

**Fig 1:**
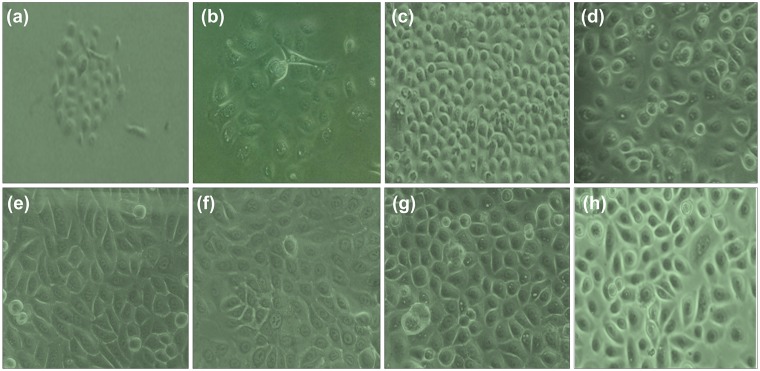
Cultures of human primary vaginal epithelial cells (hPVECS) and VK2/E6E7 cells. Different phases of growing cells of hPVECs are shown (a-d). a: Cells attached to the surface of the culture flask on day 10 (10x), b: on day 15 (40x), c: confluent cells on day 25 (10x) and d: confluent cells on day 25 (40x); e, f, g: confluent hPVECs from three different patient samples; h: confluent cultures of VK2/E6E7 cell line on day 10 (40x).

**Fig 2:**
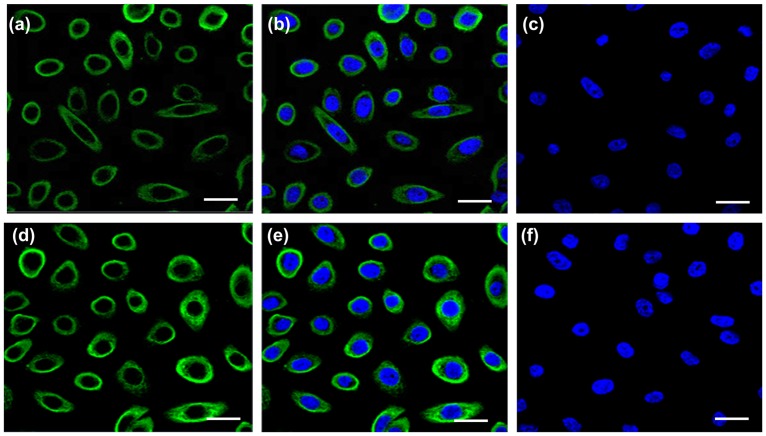
Immunofluorescence localization of cytokeratin-13. Confocal images showing cytoplasmic localization of cytokeratin-13(green) in hPVECs (a-c) and VK2/E6E7 cells (d-f). Nucleus was stained with DAPI (blue). FITC (a, d), FITC and DAPI merge (b,e) and no primary antibody controls (c,f) are shown. The figure shown is one of the representative pictures from three independent experiments (Mag. 63X).

The epithelial nature and purity of cultured human primary vaginal epithelial cells (hPVECs) are crucial for this study. The photomicrographs of different phages of growing hPVECs cultures (Fig 1a–1d), confluent hPVEC cultures of three different patients on day 25 (Fig 1e–1g), and culture of VK2/E6E7 cell line on day 10 (Fig 1h) are shown. Epithelial nature of hPVECs was tested by CK-13 immunofluroscence localization (Fig 2a–2c). The immunofluorescence results revealed that CK-13 antibody reacts only with simple, cornified and non-cornified squamous epithelial cells and pseudostratified epithelial cells. CK-13 was found to be localized in the cytoplasm of hPVECs and VK2/E6E7 cells confirming successful isolation of hPVEC population.

**Fig 3:**
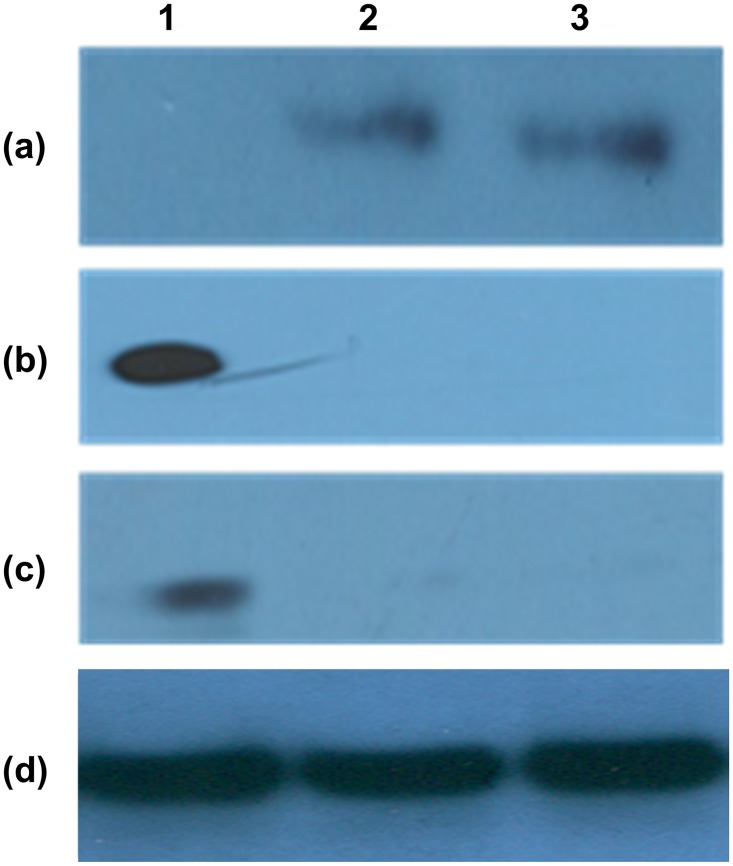
Western blot analysis of biomarker expression in hPVECs, VK2/E6E7 cells and RBCs. (a) Cytokeratin-13 (47kDa) expression: Lane-1: RBCs, lane-2: hPVECs and lane-3: VK2/E6E7 cells. (b) Solute carrier family 4 member-1 (SLC4A1) (100 kDa) expression: Lane-1: RBCs (positive control), lane-2: hPVECs, lane-3: VK2/E6E7 cells. (c)Vimentin (54 kDa) expression: Lane-1: vimentin expression in HeLa cells (positive control), Lane-2: hPVECs, Lane-3: VK2/E6E7 cells. (d) β-actin (42 kDa) expression in hPVECs (loading control). The blots shown are one of the representative pictures from three independent experiments performed on three different days.

Western blot results revealed that CK-13 was found to be expressed only by hPVECs and VK2/E6E7 cells (Fig 3, lanes-a2, a3), but not by the erythrocytes (Fig 3, lane-a1). Both hPVECs (Fig 3, lane- c2) and VK2/E6E7 cells (Fig 3, lane- c3) did not express vimentin (Fig 3, lane- c3), but HeLa cells (positive control) did show the expression (Fig 3, lane- c1). The results were further confirmed by Flow cytometric analysis (Figure A in S1 File).

#### hPVEC cultures were devoid of stromal cells and RBC contamination

**Fig 4:**
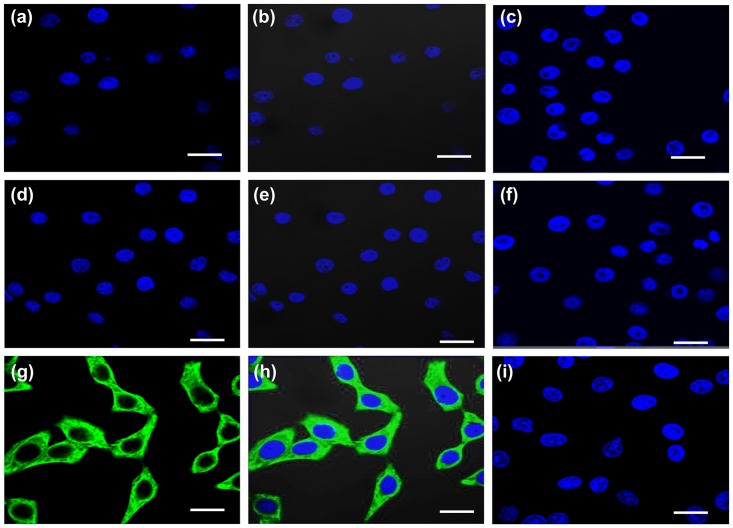
Immunofluorescence localization of vimentin. hPVECs (a-c) and VK2/E6E7 cells (d-f) did not express vimentin. Cytoplasmic localization of vimentin (green) is seen in HeLa cells (positive control) (g-i). Nucleus was stained with DAPI (blue), FITC (a, d, g), FITC and DAPI merge (b,e,h) and no primary antibody controls (c,f,i) are indicated. The figure shown is one of the representative pictures from three independent experiments (Mag. 63X).

Next, we checked whether the cultures were contaminated with stromal cells, vimentin expression was carried out by immunofluorescence (Fig 4). The results indicated that hPVECs and VK2/E6E7 cells did not stain for vimentin, suggesting the cultures were devoid of stromal cells (Fig 4a–4f). HeLa cells, which express vimentin were used as positive control (Fig 4g–4i). Further we checked whether the cultures are contaminated with RBCs using RBC specific marker protein, solute carrier family 4 member -1 (SLC4A1) by immunofluorescence. SLC4A1). The results revealed that none of the primary cells expressed SLC4A1. However, SLC4A1 expression was seen on the membranes of RBCs (Figure B in S1 File). Western blot results showed that SLC4A1 was found to be expressed only by RBCs (Fig 3, lane-b1), but not by hPVECs (Fig 3, lane-b2) or VK2/E6E7 cells (Fig 3, lane- b3). These results are in agreement with immunofluorescence data.

### LPS induced the expression of *Hb-α* and *Hb-β* mRNA in hPVECs and VK2/E6E7 cells

#### RT-PCR and qRT-PCR analysis

**Fig 5:**
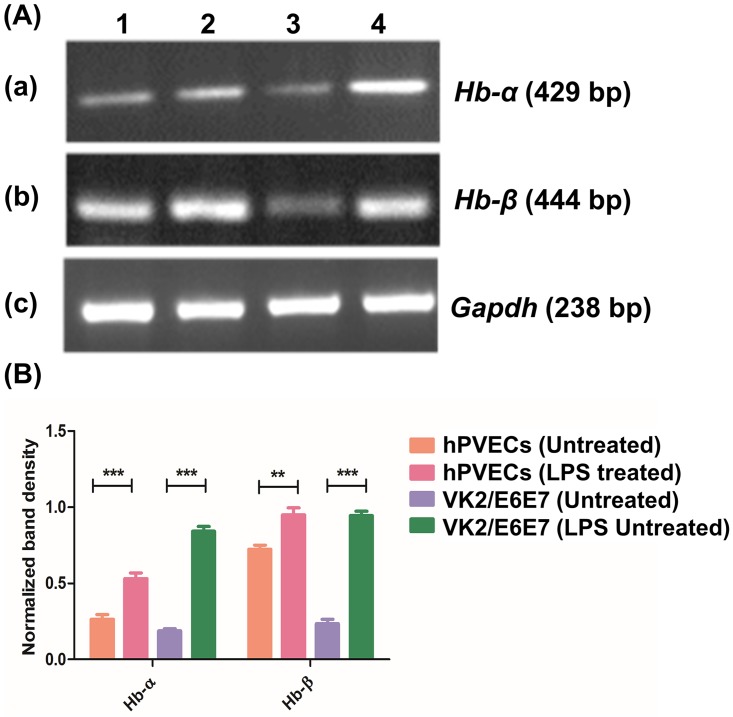
*Hb-α* and *Hb-β* expression in hPVECs and VK2/E6E7 cells. (A) RT-PCR analysis; 1: Untreated hPVECs, 2: hPVECs treated for 6 hrs with LPS (10 μg/ml), 3: Untreated VK2/E6E7 cells and 4: VK2 cells treated with LPS (10 μg/ml) for 6 hrs. Loading control, *Gapdh* (238 bp) expression in hPVECs. The gels shown are one of the representative pictures from three independent experiments performed on three different days. (B) Densitometric analysis of bands from RT-PCR amplification products of Hb-α and Hb-β mRNAs shown in figure-5A.

**Fig 6:**
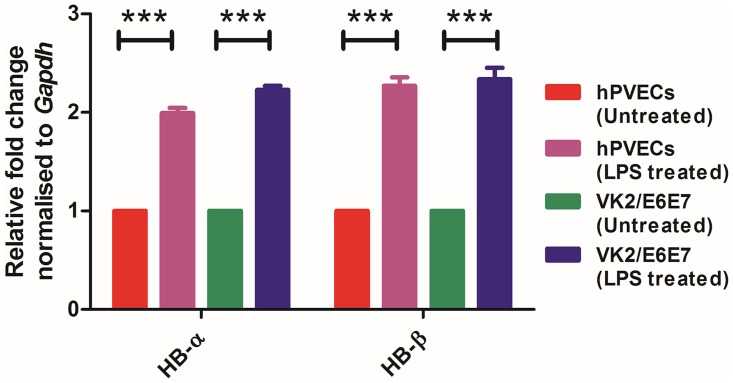
qPCR analysis of *Hb-α* and *Hb-β* expression in hPVECs and VK2/E6E7 cells before and after induction with LPS. Cells seeded at a density of 10^6^/well in 24-well plates were treated with LPS (10 μg/ml for 6 hrs). Expression of *Hb-α* and *Hb-β* was up-regulated in LPS-induced cells. Each bar represents the mean ± SD of three independent replicates (***p< 0.001 Vs Untreated).

For analyzing the expression of *Hb-α* and *Hb-β* in hPVECs and VK2/E6E7 cells, we employed RT-PCR using gene specific primers followed by sequencing of PCR products. We obtained expected PCR products having the band size of 429 bp (*Hb-α*) and 444 bp (*Hb-β*) (Fig 5A). The sequenced PCR products arranged by using Clustal ‘W’ program which showed ~80% matched with *Hb-α* and *Hb-β* mRNA sequences deposited in the NCBI nucleotide database (Figure C and Figure D in S1 File). We also investigated whether LPS could induce *Hb-α* and *Hb-β* expression in hPVECs (Fig 5A: 5a, lane 2) and VK2/E6E7 cells (Fig 5a, lane 4). Densitometric analysis of RT-PCR amplification products of Hb-α and Hb-β revealed up-regulation of *Hb-α* and *Hb-β* expression in LPS-induced cells (Fig 5B). RT-PCR results revealed that when these cells were stimulated with LPS (10 μg/ml for 6 hrs), a significant (***P<0.001) upregulation of *Hb-α* and *Hb-β* mRNAs was observed as compared to untreated cells. We further determined fold changes in the expression of *Hb-α* and *Hb-β* mRNA by qPCR, which revealed stimulation with LPS resulted induction of *Hb-α* and *Hb-β* mRNA about 2.0 folds in hPVECs and 2.2 folds in VK2/E6E7 cells (Fig 6).

#### Immunofluorescence and western blot analysis

**Fig 7:**
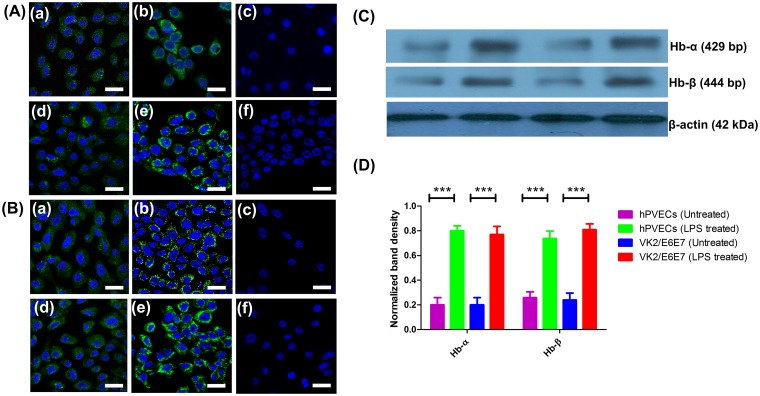
Hb-α and Hb-β expression in hPVECs and VK2/E6E7 cells at protein level. (A, B). Immunofluorescence localization of Hb-α and Hb-β. Cytoplasmic localization (green) of Hb-α (A) and Hb-β (B) was observed in hPVECs (a,b,c) and VK2/E6E7 cells (d,e,f). DAPI stained nucleus (blue). a, d: Untreated cells; b,e: LPS treated cells; c,f: Primary antibody controls did not show Hb-α and Hb-β localization. Expression was significantly up-regulated when hPVECs and VK2/E6E7 cells stimulated with LPS (10 μg/ml for 6 hrs) (b, e). The figure shown is one of the representative pictures from three independent experiments performed on three different days (Mag. 63X). (C). Western blot analysis of Hb-α and Hb-β expression in hPVECs and VK2/E6E7 cells. β-actin (42 kDa) (loading control) expression in hPVECs (1: Untreated hPVECs, 2: hPVECs induced with LPS, 3: Untreated VK2/E6E7 cells and 4: VK2/E6E7 cells induced with LPS). (D). Densitometric analysis of bands from western blot of Hb-α and Hb-β shown in Fig 7C.

The expression of Hb-α and Hb-β at protein level was examined by immunofluorescence using highly purified human Hb-α and Hb-β antibodies. One of the representative figures from three independent experiments is shown (Fig 7). The results revealed that Hb-α (Fig 7A) and Hb-β (Fig 7B) localized in the cytoplasm of the hPVECs (Fig 7 a, b and c) and VK2/E6E7 cells (Fig 7 d, e and f). Under normal condition, Hb-β expression was higher than Hb-α. (Fig 7B, a, d). When these cells stimulated with LPS (10μg/ml for 6 hrs), the expression of Hb-α and Hb-β was significantly upregulated in hPVECs (Fig 7A, b, e) and VK2/E6E7 cells ((Fig 7B, b, e) as compared to untreated cells. Cells not incubated with primary antibody were considered as negative control, which did not show any staining for Hb-α and Hb-β proteins. These results are in agreement with western blot data, wherein we demonstrated hPVECs and VK2/E6E7 cells express Hb-α and Hb-β and the expression was significantly upregulated after LPS stimulation (Fig 7C, a, b). Densitometry analysis of western blot protein bands revealed an upregulation of Hb-α (Fig 7D, a2, b4) and Hb-β (Fig 7D, b2, b4) in LPS-induced hPVECs and VK2/E6E7 cells.

### LPS-induces p65-NF-κB mRNA and Bay 11–7082 inhibited p65-NF-κB promoter activity in hPVECs and VK2/E6E7 cells

**Fig 8:**
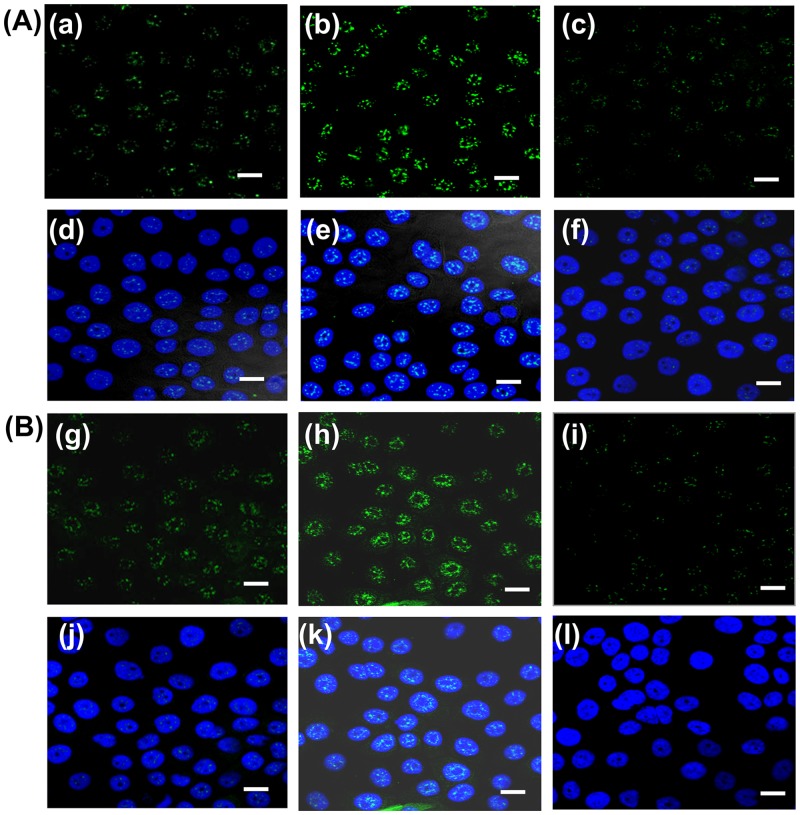
Immunofluroscence of phosphorylated p65-NF-κB expression in hPVECs and VK2/E6E7 cells. hPVECs (A) and VK2/E6E7 (B) cells were treated with LPS (10 μg/ml 6 hrs) or Bay 11–7082 (5 μM for 24 hrs). Stimulation with LPS activates p65-NF-κB expression in hPVECs (b,e) and VK2/E6E7 cells (h,k) as compared to unstimulated hPVECs (a,d) and VK2/E6E7 cells (g,j). Treatment with Bay 11–7082 attenuated NF-κB expression in hPVECs (c,f) and VK2/E6E7 cells (i,l). Nucleus was stained with DAPI (blue), p65-NF-κB was stained with FITC (green), FITC and DAPI merged (d,e,f,j,k,l). The images shown are the representative pictures of one of three identical experiments performed on three different days (Mag. X 63).

**Fig 9:**
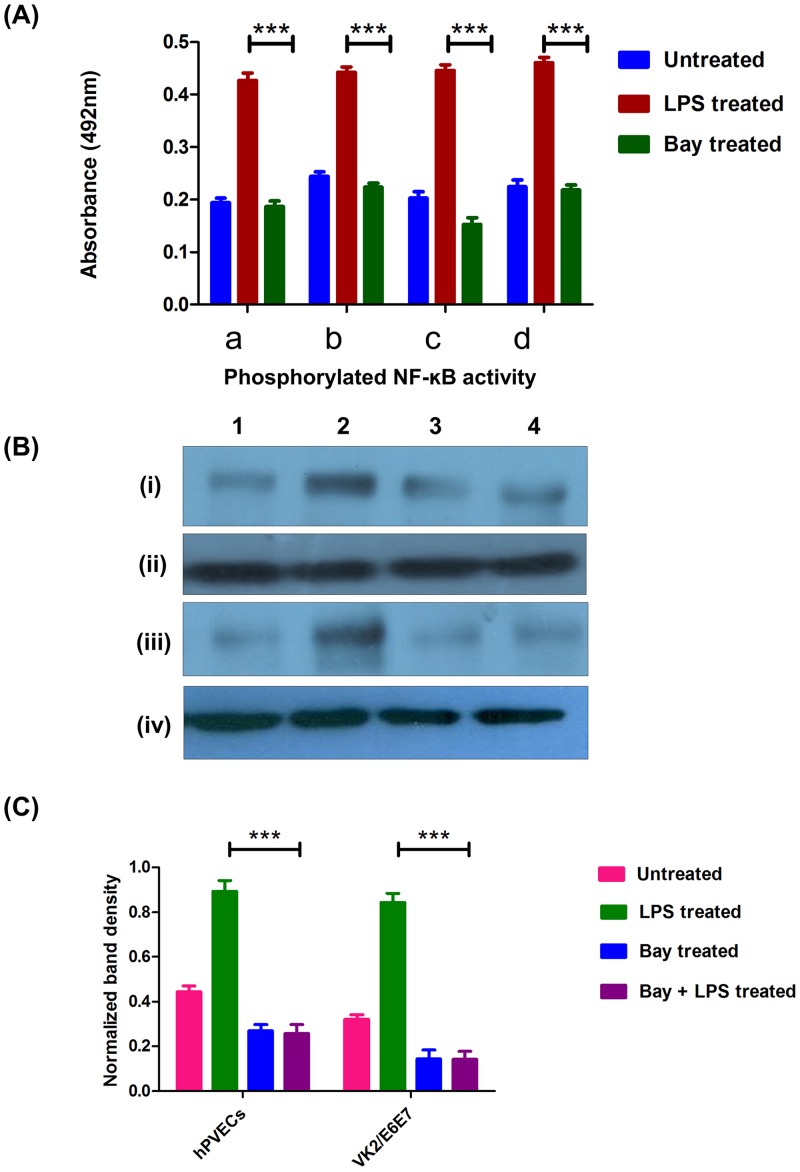
Expression of NF-*κ*B in hPVECs and VK2/E6E7 cells. (A). NF-κB levels in un-stimulated, LPS stimulated and Bay 11–7082 treated hPVECs and VK2/E6E7 cells analyzed by ELISA. Cells were seeded at a density of 10^6^/well in 24-well plates and induced with LPS (10 μg/ml for 6 hrs) or Bay-11-0782 (5 μM for 24 hrs). Levels of p65-NF-κB were up-regulated in LPS-induced cells, where as Bay-11-7082 reversed this effect (a). Values represent mean ± SD of three experiments performed on different days. Values are statistically significant (***p<0.001 over Bay 11–7082 treated cells). (B). Western blot analysis of p65-NF-κB expression in hPVECs and VK2/E6E7 cells. hPVECs (i, iii) and VK2/E6E7 cells (ii, iv)1: Untreated cells, 2: LPS induced cells, 3: Bay 11–7082 treated cells, 4: Bay 11–7082 treated cells induced with LPS. Results were normalized to the β-actin (iii, iv), which is constitutively expressed in cells and serves as an internal standard. The blots shown are the representative pictures from three independent experiments. (C). Densitometric analysis of bands from western blots of p65-NF-κB reported in Fig 9B. The levels of p65-NF-κB were up-regulated in LPS-induced cells, whereas Bay 11–7082 repressed p65-NF-κB levels.

NF-κB is a multifunctional nuclear transcription factor which regulates the function of several genes involved in innate immunity [4]. Therefore, studies were extended to examine whether LPS has any effect in the modulation of phosphorylated (p65)-nuclear factor-*κ*B (p65-NF-κB) expression. As shown in Fig 8, immunofluorescence results revealed that a significant up-regulation of p65-NF-κB after induction with LPS (10μg/ml for 6 hrs) in hPVECs (Fig 8b and 8e) and VK2/E6E7 cells (Fig 8h and 8k) as compared to the untreated cells. The nuclear expression of p65-NF-κB indicates its role in the activation of downstream signaling required for pathogen clearance. When Bay 11–7082 treated hPVECs and VK2/E6E7 cells induced with LPS, the expression of p65- NF-κB did not change and is at par with the untreated cells (Fig 8c, 8f, 8i and 8l). Similarly, ELISA (Fig 9A) and western blot (Fig 9B) results indicated that p65-NF-κB levels were significantly repressed when hPVECs and VK2/E6E7 cells were treated with Bay 11–7082 (5 μM for 24 hrs). Densitometry analysis (Fig 9C) of western blot results are in agreement with ELISA results. Thus in LPS-induced hPVECs and VK2/E6E7 cells treated with Bay 11–7082, the expression of p65-NF-κB was attenuated and the levels almost reached to baseline level, suggesting Bay 11–7082 antagonizes the effect of LPS.

### *TLR4* expression in hPVECs and VK2/E6E7 cells (RT-PCR analysis)

**Fig 10:**
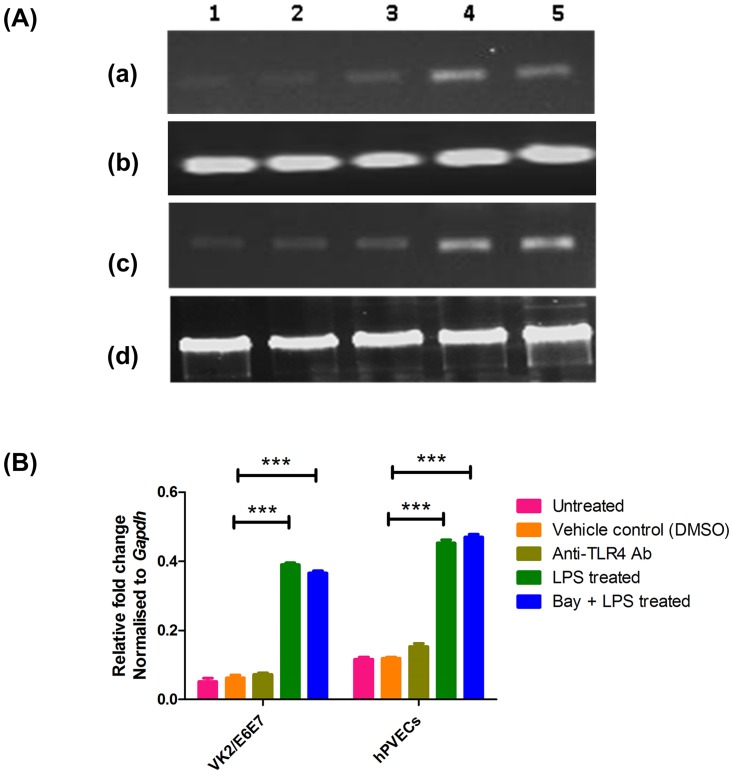
TLR4 expression in hPVECs and VK2/E6E7 cells. (A). RT-PCR analysis of TLR4 expression in VK2/E6E7 cells and hPVECs. expression of TLR4 (182 bp) in VK2/E6E7 cells (a), (c), and hPVECs (b),(d) was observed. 1: Untreated cells (control); 2: DMSO treated cells (control); 3: TLR4 antibody treated cells; 4: LPS treated cells (10 μg/ml for 6 hrs) and 5: Bay 11–7082 treated cells (5μM for 24 hrs) stimulated with LPS (10μg/ml for 6 hrs) and (c), (d) Housekeeping gene *Gapdh* (238 bp) considered as an internal standard. The gel picture shown is one of the representative pictures from three independent experiments. (B). Densitometric analysis of bands from RT–PCR amplification products of *TLR4* mRNA reported in Fig 10A. The expression of *TLR4* mRNA was up-regulated in LPS-induced cells. Bay 11–7082 treatment has no effect on the expression of TLR4 mRNA in cells induced with LPS.

To determine whether the LPS-mediated expression of *TLR4* was due to an increase in transcription, rather than to mRNA stabilization, hPVECs and VK2/E6E7 cells were treated for 24 hrs with or without Bay 11–7082 (5 μM for 24 hrs) before stimulation with LPS (10 μg/ml for 6 hrs). RT-PCR results indicated that, expression of *TLR4* mRNA (182 bp) in VK2/E6E7 cells (Fig 10A, a) and hPVECs (Fig 10A, b) was up-regulated in LPS-induced cells (Fig 10A, a4, a5, b4, b5). When Bay 11–7082 treated cells were induced with LPS, the expression of *TLR4* mRNA did not decrease (Fig 10A, a5, b5), suggesting that the LPS-induced *TLR4* mRNA expression could be due to an increased transcription. In cells treated with anti-TLR4 antibody showed absence of *TLR4* mRNA expression (Fig 10A, a3, b3). Densitometry analysis of RT-PCR bands revealed up-regulation of *TLR4* mRNA in LPS-induced cells (Fig 10B). It was further confirmed by ELISA (Figure E in S1 File).

### LPS alters the secretions of IL-6, but not IL-10 in hPVECs and VK2/E6E7 cells

**Fig 11:**
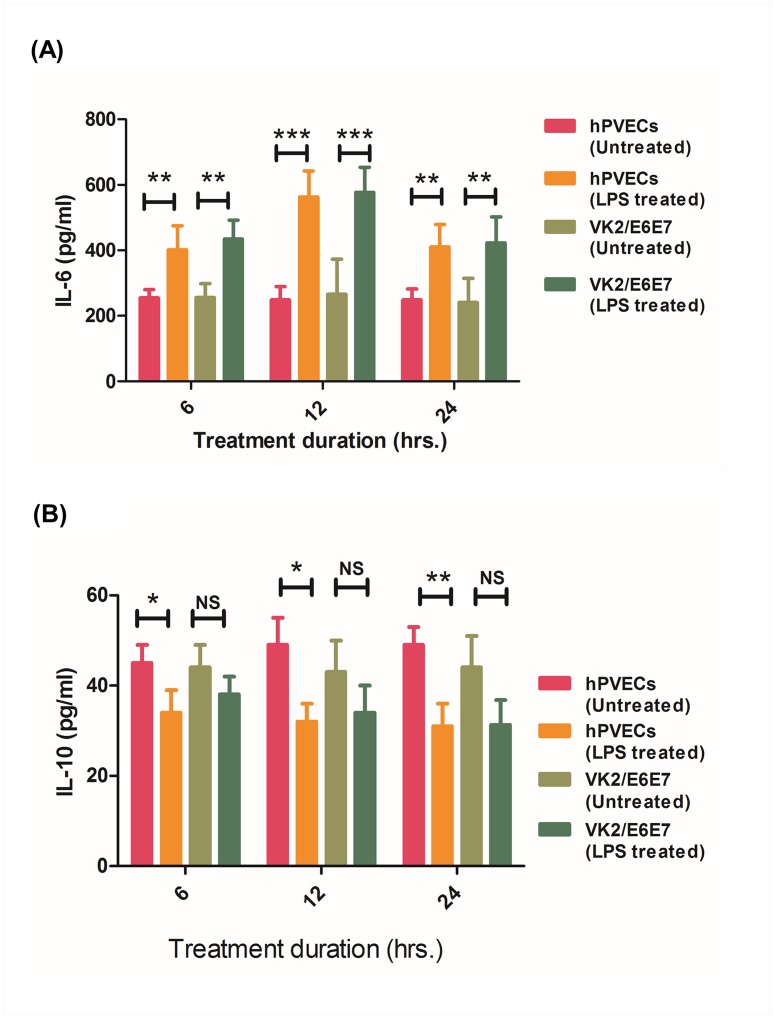
Cytokoine ELISA of hPVECs and VK2/E6E7 cells. IL-6 (A) and IL-10 (B) in the culture supernatants of un-stimulated and LPS stimulated hPVECs and VK2/E6E7 cells at indicated time points (6, 12, 24 hrs). Cells were seeded at a density of 10^6^/well in 24-well plates and treated with LPS (10 μg/ml for 6 hrs). Levels of IL-6 were up-regulated in LPS-induced cells. Values represent the mean ± SD of three independent experiments performed on different days. Level of significance (*p<0.05; **p<0.01; ***p<0.001) was calculated by students ‘t’ test followed by Bonferroni analysis.

To determine whether hPVECs and VK2/E6E7 cells synthesize IL-6 and IL-10 proteins, culture supernatants of untreated and LPS stimulated cells collected after 6, 12 and 24 hrs were analyzed by sandwich ELISA using the Kit for IL-6 and IL-10. As shown in Fig 11A and 11B, hPVECs and VK2/E6E7 cells constitutively secreted low levels of IL-6 and IL-10. However, when these cells were stimulated with LPS (10 μg/ml) for 6, 12 and 24 hrs, IL-6 levels were significantly increased after 12 hrs as compared to 6 and 24 hrs (Fig 11A). In contrast, IL-10 levels were decreased albeit non-significantly after LPS stimulation in both hPVECs and VK2/E6E7 cells (Fig 11B).

### P65-NF-κB repression attenuates LPS-induced expression of *Hb-α* and *Hb-β* genes in hPVECs and VK2/E6E7 cells

**Fig 12:**
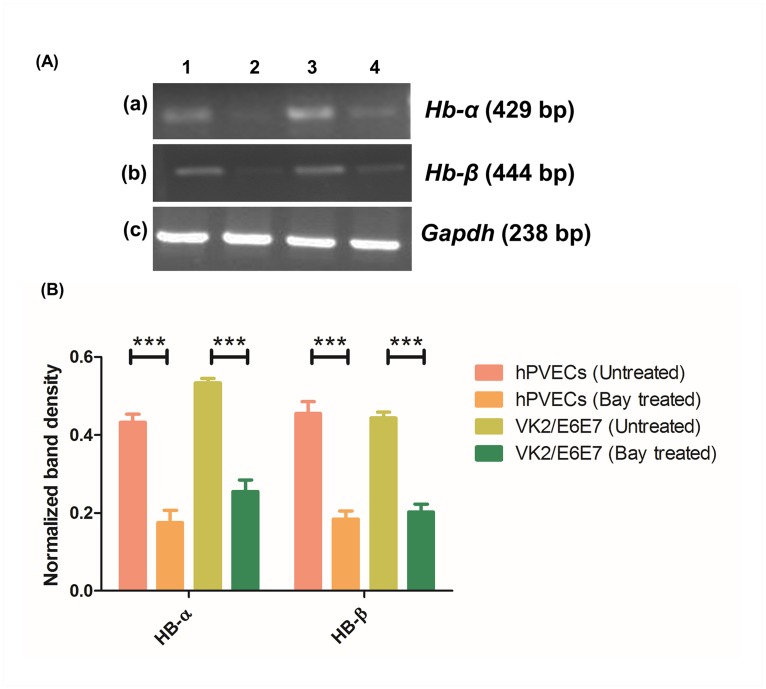
RT-PCR analysis of *Hb-α* and *Hb-β* in presence of LPS and Bay 11–7082. (A) *Hb-α* (429 bp) (a) and *Hb-β* (444 bp) (b) expression was observed in hPVECs (1,2) and VK2/E6E7 cells (3,4) before and after the treatment with LPS (10 μg/ml for 6 hrs) and Bay 11–7082 (5μM for 24 hrs). LPS-induced (a1, a3, b1, b3), Bay 11–7082 treated (a2, a4, b2, b4) are shown. Expression of *Hb-α* and *Hb-β* was up-regulated in LPS-induced cells, whereas Bay 11–7082 attenuated this expression (c) *Gapdh* (238 bp) was used as loading control. The gels shown are the representative pictures from three independent experiments. (B). Densitometric analysis of RT-PCR bands shown in Fig 12A. Expression of both *Hb-α* and *Hb-β* was elevated in LPS-stimulated cells. In contrast, Bay 11–7082 attenuated the expression of *Hb-α* and *Hb-β*.

Attempts were made to check whether blocking of p65-NF-κB effects the expression of *Hb-α* and *Hb-β* in hPVECs and VK2/E6E7 cells. Based on the initial standardization with respect to dose and time to elicit maximum inhibition of NF-κB response, we choose 5 μM dose and 24 hrs treatment duration. RT-PCR results revealed that expression of *Hb-α* (429 bp) and *Hb-β* (444 bp) transcripts was significantly repressed after the treatment with Bay 11–7082 (Fig 12A). Relative expression was calculated and normalized to the values of *Gapdh*. Intensities of the RT-PCR bands were quantified using Quantity-one software. *Hb-α* and *Hb-β* expression decreased by 50–60% in Bay 11–7082 treated cells (Fig 12B).

### LPS-induced expression of *hBD-1* mRNA

**Fig 13:**
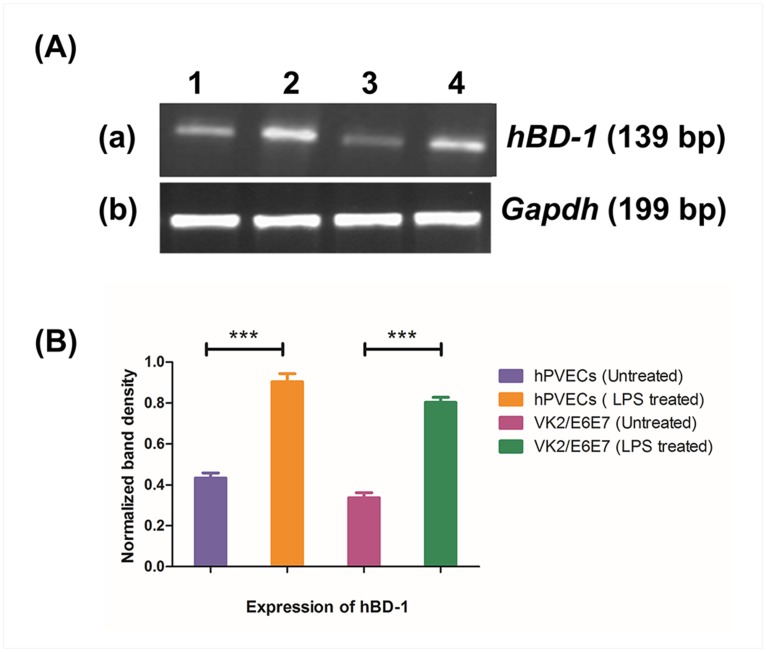
RT-PCR analysis of *human-β defensin-1* (*hBD-1*). (A) mRNA expression of *hBD1* (139bp) was observed in un-stimulated (a1, a3), LPS stimulated (a2, a4) in hPVECs (a1, a2) and VK2/E6E7 cells (a3, a4). Cells were seeded at a density of 10^6^/well in a 24-well plates and treated with LPS (10 μg/ml for 6 hrs). Expression of *hBD1* mRNA was up-regulated in LPS-induced cells. Representative image of RT-PCR analysis of *hBD-1* mRNAs expression is shown. *Gapdh* confirmed roughly equivalent loading of RNA samples. 1: hPVECs (Unstimulated); 2:hPVECs induced with LPS; 3:VK2/E6E7 cells (Unstimulated); 4:VK2/E6E7 cells stimulated with LPS (10μg/ml). (B). Densitometric analysis of bands from RT-PCR amplification products of *hBD1* mRNA reported in Figure 13A. Expression of *hBD-1* was increased in LPS-stimulated hPVECS and VK2/E6E7 cells.

Epithelial cells of vaginal mucosal surface are critical interfaces interacting with the external environment and are the first to come in contact with pathogens, suggesting hPVECs play an important role as the host innate immune defense [30]. However, the immunological responses of these cells upon stimulation with LPS are not known. Therefore, we examined whether the upregulated expression of IL-6 is associated with cellular immune functions related to the defense pathway. For this, we chose hBD-1, a known marker for epithelial cell protection against pathogens. The expression of *hBD-1* was determined at mRNA level in hPVECs and VK2/E6E7 cells before and after induction with LPS (10 μg/ml for 6 hrs). The RT-PCR results revealed that a significant up-regulation of *hBD-1* mRNA (139 bp) expression after LPS- induction in hPVECs and VK2/E6E7 cells (Fig 13A). Relative expression was calculated and normalized to the values of *Gapdh* (238 bp) and found ~50% increase in *hBD-1* expression after LPS stimulation (Fig 13B). Collectively, these results suggest that hBD-1 may be a component of the endogenous anti-inflammatory defense system, protecting vaginal epithelial cells against inflammation/infection induced damage.

### Prediction of NF-κB binding sites on Hb-α promoter

We analyzed human Hb-α promoter region and identified potential NF-κB transcription factor binding sites upstream of the TSS (Figure F in S1 File). The ALGGEN—PROMO software analysis indicated presence of NF-κB binding site within region between nt-115 to-106 of Hb-α promoter.

### Effect of LPS on Hb-α transcription factor binding

**Fig 14:**
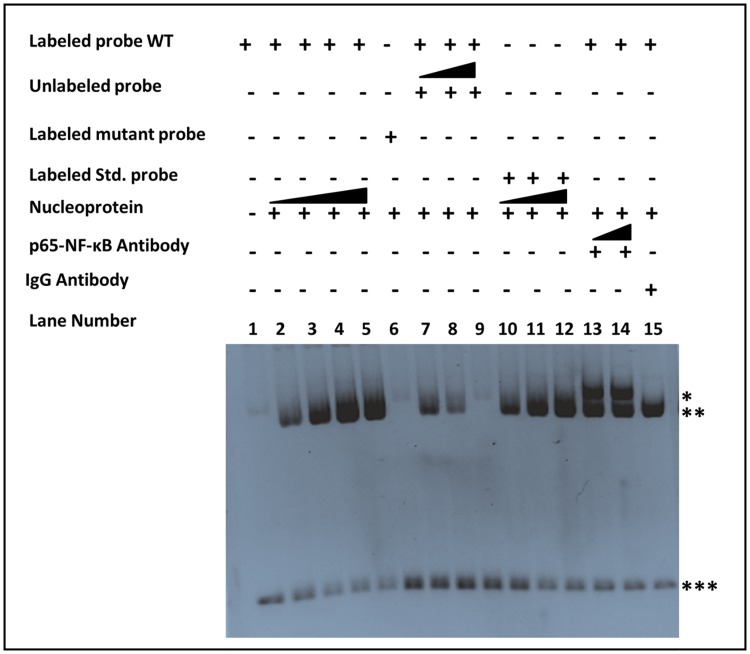
Electrophoretic mobility shift assay (EMSA) of Hb-α with LPS. EMSA was performed using oligonucleotide probes corresponding to NF-κB regulatory elements. Nuclear extract from LPS stimulated hPVECs was used. Nucleoprotein containing NF-κB was incubated with a fragment encompassing nt-115 to -106 bp upstream of Hb-α promoter start site-2. We observed a strong DNA binding to NF-κB protein with LPS treatment. Presence of super-shift bands in the presence of the p65- NF-κB antibody demonstrating the specific binding. (***: denotes unbound DIG oxygenase labelled DNA; 1- Only wild type labelled probe; 2- Wild type labelled Probe + Protein (1μg); 3- Wild type labelled Probe + Protein (+2μg); 4- Wild type labelled Probe + Protein (++ 4μg); 5- Wild type labelled Probe + Protein (+++ 5μg); 6- Mutant labelled Probe + Protein (1μg); 7- Wild type labelled Probe + unlabelled Probe 1x + Protein (1μg); 8- Wild type labelled Probe + unlabelled Probe 2x + Protein (1μg); 9- Wild type labelled Probe + unlabelled Probe 4x + Protein (1μg); 10- Standard labelled Probe 1x + Protein (1ug); 11- Standard labelled Probe 2x + Protein (1μg); 12- Standard labelled Probe 4x + Protein (1μg); 13- Wild type labelled Probe + Protein (1μg) + p65- NF-κB antibody(1x); 14- Wild type labelled Probe + Protein (1μg) + p65-NF-κB antibody(2x) and 15- Wild type labelled Probe + Protein (1μg) + anti rabbit IgG(1x). (*:NF-κB -antibody- NF-κB -DNA complexes and **: NF-κB -DNA complexes.

As discussed above, stimulation of VK2/E6E7 cells with LPS induced the expression of Hb-α mRNA and protein. qPCR results confirmed a marked increase of Hb-α mRNA expression by LPS. It is known that LPS binds to TLR4 and activate NF-κB, which trans-activates various other genes. Therefore, attempts were made to determine the possible interaction of NF-κB with Hb-α promoter after LPS stimulation in hPVECs and VK2/E6E7 cells by EMSA. We used double-stranded oligonucleotide probes corresponding to putative binding sites of NF-κB present on the Hb-α promoter (nt-115 to -106) identified by *in-silico* analysis using ALGGEN-PROMO software. These probes were DIG-labeled. Incubation of nuclear protein extracts from LPS-induced VK2/E6E7 cells and hPVECs with Hb-α promoter oligonucleotides resulted in a sitespecific binding (Fig 14). With the addition of higher concentration of protein resulted in increased specific NF-κB binding activity (lanes 2, 3, 4 & 5). Addition of 2 and 4-fold excesses of specific cold competitors (unlabeled NF-κB oligonucleotides) decreased the binding of nuclear proteins to NF-κB (lanes 7, 8 & 9) oligonucleotides. No specific complex was formed with mutated probe (lane 6). Binding of NF-κB to the control standard probe was observed with its increasing concentration (lanes 10, 11, 12). To ascertain the specificity of the nuclear proteins bound to NF-κB site, NF-κB antibody super-shift assay was performed. A prominent single super-shift band was observed when nuclear extracts were incubated with anti-NF-κB antibody (lane 13 & 14), but not with rabbit control IgG (lane 15). Thus, these results confirmed that NF-κB bind to the Hb-α promoter in a sequence-specific manner.

### Validation of NF-κB to Hb-α promoter in hPVECs stimulated with LPS by ChIP assay

**Fig 15:**
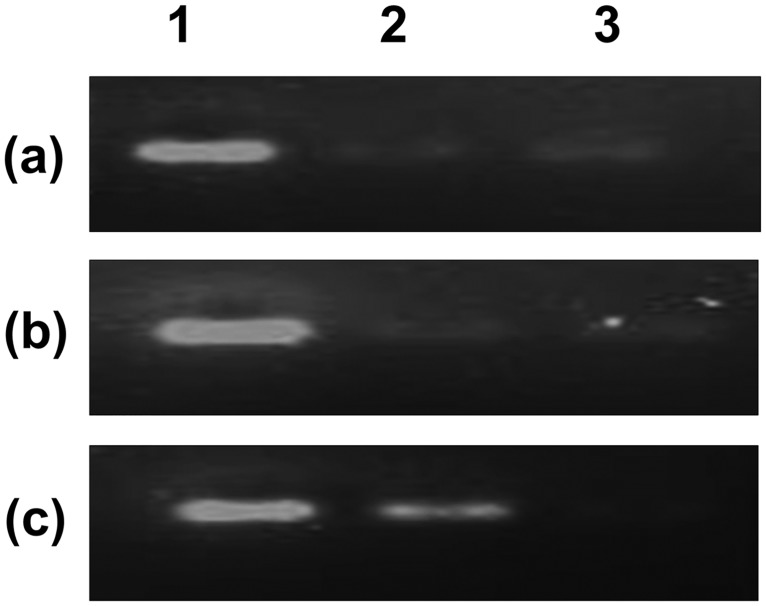
Verification of NF-κB interactions with the binding sites in the Hb-α promoter by Chromatin immune precipitation assay (ChIP). Cross-linked and sheared chromatin from hPVECs was immune-precipitated with p65- NF-κB antibody and analysed by RT-PCR. The results revealed that NF-κB efficiently interacted with the binding sites present in the Hb-α promoter (a: untreated; b: LPS treated (10 μg/ml for 2 hrs); c: LPS treated for 24 hrs; 1: Input sample from control; 2: p65- NF-κB antibody pull down from control and LPS treated (b,c); 3: IgG antibody pull down from control (a) and LPS treated (b, c).

We confirmed NF-κB binding to the Hb-α promoter in hPVECs using chromatin immuno-precipitation (ChIP) assay (Fig 15). EMSA experiment helped us to establish loss or enhancement of transcription factor NF-κB association with active elements within the Hb-α promoter.

hPVECs were treated with LPS and fixed with formaldehyde to stabilize DNA–protein complexes. After shearing DNA by sonication, chromatin fragments were immune-precipitated with antibody against p65 NF-κB subunit. Co-immuneprecipitated DNA was used as template for PCR with pairs of primers flanking NF-κB sites in the Hb-α promoter. The results show that after 2 hrs of LPS treatment, no significant expression was observed as compared to 24 hrs of LPS treatment (Lane 2). Control IgG antibody pull down did not show any PCR amplification (Lane 3). The input in all three conditions showed amplification for NF-κB binding site. Thus, our results revealed increased amplification of Hb-α promoter DNA co-immunoprecipitated by anti-p65 antibody, but not by pre-immune serum, suggesting LPS-induced binding of p65 complex to the NF-κB sites of the Hb-α promoter.

## Discussion

The mucosal barrier plays an essential role in preventing infection by pathogens in the female reproductive tract (FRT). In this study, we established the culture of primary vaginal epithelial cells (PVECs) from human vaginal tissue and confirmed the presence of epithelial features, such as a cobble-stone-like morphology, tight connections and clear boundaries. The epithelial nature of these cells was confirmed by epithelial cell specific marker, cytokeratin 13 (CK-13) [37] through immunofluorescence, western blot and flow cytometric analysis. The cytoplasmic localization of CK-13 was consistent with the role of keratins as intracellular intermediate filaments. The contamination of hPVECs with stromal cells or mesenchymal cells and RBCs was also ruled out by analyzing vimentin and SLC4A1 expression. As expected, the expression of vimentin and SLC4A1 was not observed in hPVECs and VK2/E6E7 cells, suggesting cultures are free from these contaminations.

The expression of Hb-α and Hb-β proteins was evaluated by immunofluorescence and western blot, which revealed constitutive expression by hPVECs and the expression was at par with VK2/E6E7 cells, suggesting both Hb-α and Hb-β were indeed synthesized by these cells. It has been reported that Hb-α and Hb-β expressed by erythrocytes and silenced in non-erythroid cells [7, 17]. In erythroid cells, expression is critical for O_2_ transport, ameliorating hypoxia induced oxidative stress, [38], but little is known of their functions in non-erythroid cells. We believe that, Hb subunits may have different functional significance in different cell types. Further studies are needed to understand the complete structure of Hb expressed in non-erythroid cells. At present, it is not known whether Hb synthesized by vaginal cells have any role such as protection of vaginal cells from invading pathogens. Therefore, we determined the effect of gram negative bacterial membrane protein, LPS on the production of Hb-α and Hb-β in hPVECS and compared them with the production of VK2/E6E7 cells. The results revealed that Hb-α and Hb-β expression was remarkably upregulated after induction with LPS, attributing they may have a role in vaginal inflammation/infection, since Hb is known to act as a precursor for the synthesis of antimicrobial peptides (AMPs) [39]. Our observations are in agreement with the results reported in mouse macrophages [11] and rat kidney cells [17], wherein the authors reported the synthesis of Hb is essential to maintain cell viability against inflammation. In murine macrophages, treatment with LPS led to the activation of *Hb-α* gene [11]. However, the mechanism by which LPS influence the expression of Hb-α and Hb-β and the pathway involved is still not clear. Recent reports suggest that LPS induces the expression of various transcription factors involved in cellular immune responses. Therefore, we speculate that NF-κB pathway might be involved in the modulation of Hb-α and Hb-β expression via TLR-4-NF-κB downstream signaling. To know this, we determined the role of NF-κB and TLR4 in the induction of *Hb-α* and *Hb-β* genes by LPS.

To determine binding sequences of NF-κB on the promoter region of the human Hb-α gene and to understand the transcriptional regulation of Hb-α and Hb-β synthesis, we used EMSA and ChIP assays. The Hb-α promoter region was analyzed for possible regulatory elements which could account for its induction by LPS. Using online -ALGGEN-PROMO software, we analyzed Hb-α promoter sequence and identified putative NF-κB binding motif in the promoter region of Hb-α. NF-κB binding site within the Hb-α promoter suggests induction of Hb-α gene transcription by LPS. To confirm these predicted analysis, we performed EMSA, which showed NF-κB effectively bound to wild-type oligonucleotides, but not to the mutated ones. In addition, p65 antibodies induced super shift of the probes, suggesting NF-κB promote the transcription of Hb-α by binding to the Hb-α promoter in hPVECs. The EMSA independently confirmed the association of NF-κB with Hb-α promoter. Presence of binding site in Hb-α for NF-κB may be implicated as important factors mediating the role of Hb-α in response to infection induced processes [40].

Next, we performed ChIP assay and confirmed the EMSA results, which suggests an interaction of p65-NF-κB with Hb-α promoter following LPS stimulation in hPVECs. This association was not observed in untreated hPVECs. Hb-α over-expression has been reported recently in several types of cancer including vaginal cancer [20]. To the best of our knowledge, ours is the first report that identified Hb-α as a direct downstream target of p65-NF-κB. These results also provide substantial evidences for the involvement of p65-NF-κB in Hb-α gene regulation at the genomic level. However, its relation to inflammation/infection is still unclear and warrant further functional studies.

**Fig 16:**
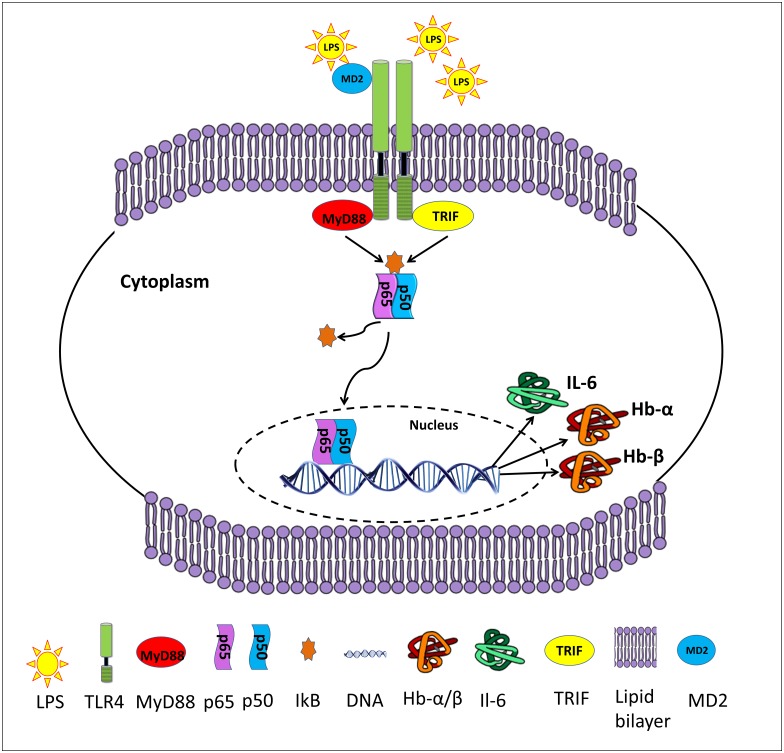
Hypothesized mechanism of Hb-α and Hb-β expression and their role in the function vaginal cells. Stimulation of hPVECs with LPS up-regulates the expression TLR4, NF-κB, Hb-α, Hb-β and pro-inflammatory cytokine (e.g. IL-6). The co-receptor MD2 triggers interactions between the cell surface domain of TLR4 and LPS. Activated TLR4 interacts with cytoplasmic adaptor molecules MyD88 or TRIF which subsequently activates IKK complex. The IKK complex induces phosphorylation of IκB and its subsequent degradation liberates NF-κB and allows it to translocate into the nucleus where it can induce target gene expression including Hb-α, Hb-β and IL-6 etc and may contribute to pathogen clearance by enhancing local host immune responses in hPVECs. This figure represents the best-fit model for these effects.

It is well known that LPS activates NF-κB via TLR-4 and contributes to the inflammatory response in various cells [41]. Therefore, we investigate whether TLR-4/ NF-κB signaling axis is involved in Hb-α and Hb-β expression in hPVECs and VK2/E6E7 cells. Our results indicated that NF-κB is active in hPVECs and VK2/E6E7 cells under normal physiological conditions and participates in the regulation of Hb-α and Hb-β expression. Here we report the new findings that Hb-α and Hb-β expression was blocked by the inhibition of p65-NF-κB activity, which was activated by LPS. Thus, we speculate that LPS enhance Hb-α and Hb-β expression via promoting p65-NF-κB downstream pathway (Fig 16).

To confirm the above observations, we examined whether the induction of Hb-α and Hb-β expression by LPS will be suppressed by inhibitor of NF-κB (Bay 11–7082). For this hPVECS and VK2/E6E7 cells were treated with Bay 11–7082 (5 μM for 24 hrs) and observed significant inhibition of Hb-α and Hb-β expression. These data provide an insight into mechanisms by which Bay 11–7082 may paradoxically inhibit the action of LPS and repress the synthesis of Hb-α and Hb-β. Also, decreased expression of either p65-NF-κB or TLR4 or both significantly abolished the production of *hBD-1* gene. Therefore, we hypothesize that p65-NF-κB might be directly involved in the modulation of Hb-α and Hb-β expression in hPVECS and VK2/E6E7 cells. Taken together, our results from the *in vitro* stimulation model reveal that there exists the pathway in hPVECs via TLR4/p65-NF-κB axis in response to LPS stimulation. We thereby believe that the use of hPVECs along with VK2/E6E7 cells enabled us to predict the source of Hb-α and Hb-β produced in the enriched hPVEC population. Accordingly, since the Hb-α and Hb-β was produced by both hPVECs and VK2/E6E7 cells and hPVEC population were 100% pure, our data suggest that the Hb-α and Hb-β were indeed epithelial cell derived.

The vaginal epithelium itself appears to play a key role in the upregulation of host immune defense by recognizing and subsequently responding to invading pathogenic threats by secreting pro-inflammatory cytokines and chemokines and AMPs [42, 43]. These molecules play an important role in maintaining vaginal homeostasis under steady-state and inflammatory conditions. Therefore, we sought to determine whether IL-6 and IL-10 are produced by hPVECs and VK2/E6E7 cells in response to LPS stimulation. IL-6 and IL-10 are typical pleiotropic cytokines, which modulates a variety of physiological events [44]. IL-6 is a pro-inflammatory cytokine implicated in inflammation activities. In contrast, IL-10 is an immune-regulatory cytokine plays a key role in protecting the host from infections. Our results indicated that IL-6 and IL-10 protein were constitutively expressed by hPVECs albeit in low level and IL-6 levels were increased following LPS stimulation. In contrast, no significant change in IL-10 levels were observed, this situation may promote pathogen clearance, and provide additional evidence for the contribution of hPVECs and VK2/E6E7 cells to vaginal defense against LPS-induction.

Taken together, the data suggest that hPVECs and VK2/E6E7 cells are similar in their capacity to produce Hb-α, Hb-β and cytokines. LPS-induces synthesis of Hb-α and Hb-β in hPVECs VK2/E6E7 cells. The transcriptional activation of TLR4 by LPS occurs through the mediation of NF-κB dependent pathway. The hPVECs and VK2/E6E7 cells respond to LPS and may contribute to the immune response at mucosal site. However, there are some limitations to this study. The studies performed here are based on *in vitro* culture conditions and may not reflect truly the activity of the cells in the intact vaginal mucosal microenvironment. Future *in vivo* studies using animal models are essential to support our *in vitro* results. In summary, this is the first comprehensive evaluation of Hb-α, Hb-β production by hPVECs and VK2/E6E7 cells in response to LPS. Our study demonstrated that p65-NF-κB is directly involved in the transcription regulation of Hb-α in hPVECs. The activated p65-NF-κB then drives the transcription of Hb-α to attenuate inflammation and or infection or both. Thereby, TLR-4/NF-κB signaling cascade is an attractive therapeutic target for vaginal inflammation and infections.

## Supporting information

S1 File Supporting file containing figures and tables. (PDF)
