# Supplementary material for: Expression of hemoglobin-α and β subunits in human vaginal epithelial cells and their functional significance
Source: PLoS One. 2017 Feb 8;12(2):e0171084. doi: 10.1371/journal.pone.0171084 (PMC5298339; doi:10.1371/journal.pone.0171084)
Supplement: S1 File — (PDF) [file pone.0171084.s001.pdf]

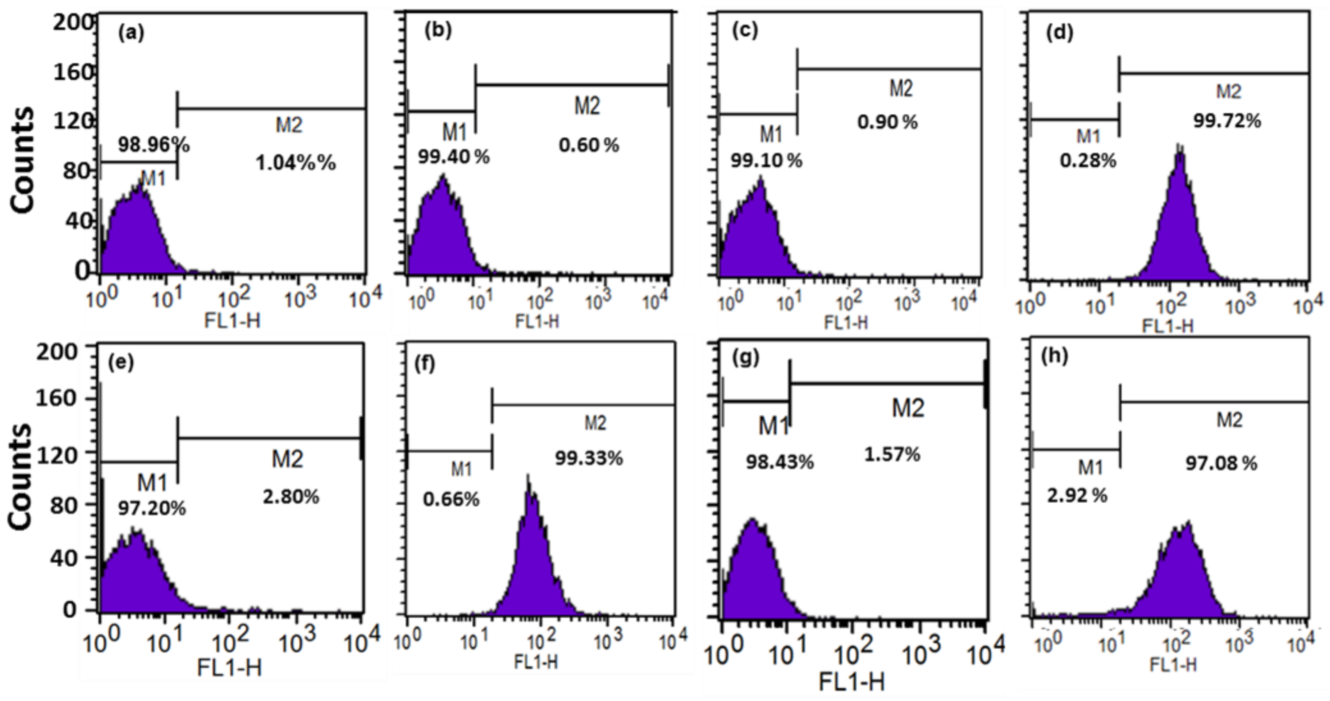

Figure A. Flow cytometry analysis of vimentin (a-d) and cytokeratin-13 (e-h) expression in hPVECs (a,b,c,e,f,g) and HeLa cells (d) (positive control for vimentin). Cells were labelled with anti-vimentin and anti-cytokeratin-13 antibodies. Comparison of CK-13 and vimentin were made by comparing fluorescence in the M1 and M2 channels. hPVECs did not show vimentin expression (b) as compared to HeLa cells (d). Cytokeratin expression was observed in hPVECs (f) and VK2/E6E7 cells (h). The figures shown are the representative pictures from three independent experiments are shown. Primary antibody controls for vimentin expression in hPVECs (a) and HeLa cells (c). Primary antibody controls for CK-13 expression in hPVECs (e) and VK2/E6E7 cells (g) are shown (FLI-H: log fluorescence intensity).

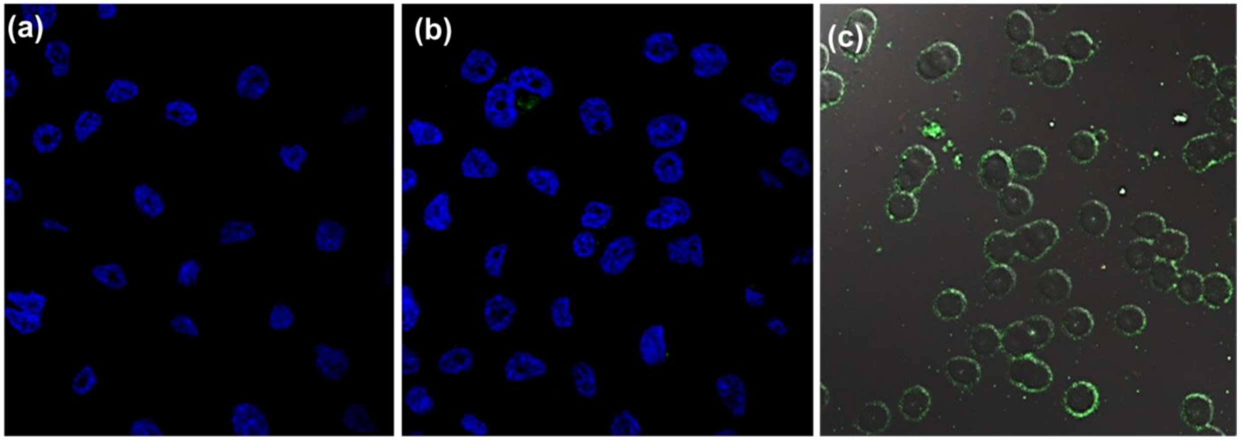

Figure B. Immunofluorescence localization of SLC4A-1 in hPVECs (a), VK2/E6E7 cells (b) and RBCs (c). SLC4A1 expression was seen on the cell membrane of RBCs (arrows). No expression was seen in hPVECs (a) and VK2/E6E7 cells (b). Nuclei of hPVECs (a), VK2/E6E7 cells stained with DAPI. Figure shown (Mag. 40X) is the representative picture from three independent experiments (a, b, c: FITC + DAPI merge).

```
clustalw.aln

CLUSTAL 2.1 multiple sequence alignment

gi|VK2/E6E7cells|_HBA__mRNA      --------------------------------------------------
gi|NM_000558.4|_HBA1__mRNA       CATAAACCCTGGCGCGCTCGCGGCCCGGCACTCTTCTGGTCCCCACAGAC

gi|VK2/E6E7cells|_HBA__mRNA      --------------------------------------------------
gi|NM_000558.4|_HBA1__mRNA       TCAGAGAGAACCCACCATGGTGCTGTCTCCTGCCGACAAGACCAACGTCA

gi|VK2/E6E7cells|_HBA__mRNA      CCGCTGCCCGGTGTA-GGTCGGCGCGCACGCTGGCGAGTATGGTGCGGAG
gi|NM_000558.4|_HBA1__mRNA       AGGCCGCCTGGGGTAAGGTCGGCGCGCACGCTGGCGAGTATGGTGCGGAG
                                   ** *** ** *** **********************************

gi|VK2/E6E7cells|_HBA__mRNA      GCCCTGGAGAGGATGTTCCTGTCAATCCCCACCACCAAGACCTACTTCCC
gi|NM_000558.4|_HBA1__mRNA       GCCCTGGAGAGGATGTTCCTGTCCTTCCCCACCACCAAGACCTACTTCCC
                                 ***********************  *************************

gi|VK2/E6E7cells|_HBA__mRNA      GCACTTCGACCTGAGCCACGGCTCTGCCCAGGTTAAGGGCCACGGCAAGA
gi|NM_000558.4|_HBA1__mRNA       GCACTTCGACCTGAGCCACGGCTCTGCCCAGGTTAAGGGCCACGGCAAGA
                                 **************************************************

gi|VK2/E6E7cells|_HBA__mRNA      AGGTGGCCGACGCGCTGACCAACGCCGTGGCGCACGTGGACGACATGCCC
gi|NM_000558.4|_HBA1__mRNA       AGGTGGCCGACGCGCTGACCAACGCCGTGGCGCACGTGGACGACATGCCC
                                 **************************************************

gi|VK2/E6E7cells|_HBA__mRNA      AACGCGCTGTCCGCCCTGAGCGACCTGCACGCGCACAAGCTTCGGGTGGA
gi|NM_000558.4|_HBA1__mRNA       AACGCGCTGTCCGCCCTGAGCGACCTGCACGCGCACAAGCTTCGGGTGGA
                                 **************************************************

gi|VK2/E6E7cells|_HBA__mRNA      CCCGGTCAACTTCAAGCTCCTAAGCCACTGCCTGCTGGTGACCCTGGCCG
gi|NM_000558.4|_HBA1__mRNA       CCCGGTCAACTTCAAGCTCCTAAGCCACTGCCTGCTGGTGACCCTGGCCG
                                 **************************************************

gi|VK2/E6E7cells|_HBA__mRNA      CCCACCTCCCCGCCGAGTTCACCCCTGCGGTGCACGCCTCCCTGGACAAG
gi|NM_000558.4|_HBA1__mRNA       CCCACCTCCCCGCCGAGTTCACCCCTGCGGTGCACGCCTCCCTGGACAAG
                                 **************************************************

gi|VK2/E6E7cells|_HBA__mRNA      TTCCTGGCTTCTGTGAGCACCGTGCTGACCTCCAAATACCGTTAAAAT--
gi|NM_000558.4|_HBA1__mRNA       TTCCTGGCTTCTGTGAGCACCGTGCTGACCTCCAAATACCGTTAAGCTGG
                                 *********************************************  *

gi|VK2/E6E7cells|_HBA__mRNA      ----TACTTATCCACTCCAAAAGCAGCGAGAGAAGCTTTTTA------TT
gi|NM_000558.4|_HBA1__mRNA       AGCCTCGGTGGCCATGCTTCTTGCCCCTTGGGCCTCCCCCCAGCCCCTCC
                                     *   *  ***  *     **  *  * *   *     *

gi|VK2/E6E7cells|_HBA__mRNA      TTATATATCTCCGCGCGCGGCCCGGGGAAAAACAAAAAACCTTCT-----
gi|NM_000558.4|_HBA1__mRNA       TCCCCTTCCTGCACCCGTACCCCCGTGGTCTTTGAATAAAGTCTGAGTGG
                                 *    *  ** * * **   *** * *       ** **  *

gi|VK2/E6E7cells|_HBA__mRNA      ---------------------------
gi|NM_000558.4|_HBA1__mRNA       GCGGCAAAAAAAAAAAAAAAAAAAAAA
```

```
CLUSTAL 2.1 Multiple Sequence Alignments


Sequence type explicitly set to DNA
Sequence format is Pearson
Sequence 1: gi|VK2/E6E7cells|_HBA__mRNA   482 bp
Sequence 2: gi|NM_000558.4|_HBA1__mRNA    627 bp
Start of Pairwise alignments
Aligning...



Sequences (1:2) Aligned. Score: 83.61
Guide tree file created:   [clustalw.dnd]

There are 1 groups
Start of Multiple Alignment

Aligning...
Group 1: Sequences:   2      Score:8022
Alignment Score 3278

CLUSTAL-Alignment file created  [clustalw.aln]
```

Figure C. Clustal W alignment of sequenced Hb-α PCR product. Hb-α (HBA1) sequence is having the accession number, NM_000518.4

```
clustalw.aln

CLUSTAL 2.1 multiple sequence alignment

gi|VK2/E6E7cells|_HBB__mRNA         --------------------------------------------------
gi|ref|NM_000518.4|_HBB__mRNA       ACATTTGCTTCTGACACAACTGTGTTCACTAGCAACCTCAAACAGACACC

gi|VK2/E6E7cells|_HBB__mRNA         -------------------------------CTACGTTTGCC--GTCGGG
gi|ref|NM_000518.4|_HBB__mRNA       ATGGTGCATCTGACTCCTGAGGAGAAGTCTGCCGTTACTGCCCTGTGGGG
                                                                   *      ****  ** ***

gi|VK2/E6E7cells|_HBB__mRNA         CAAGT--GACGTGGATGA-GTTGGTGGTGAGGCCCTGGGCAGGCTGCTGG
gi|ref|NM_000518.4|_HBB__mRNA       CAAGGTGAACGTGGATGAAGTTGGTGGTGAGGCCCTGGGCAGGCTGCTGG
                                    ****    ********** *******************************

gi|VK2/E6E7cells|_HBB__mRNA         TGGTCTACC-TTGGACCCAGAGGTTCTTTGAGTCCTTTGGGGATCTGTCC
gi|ref|NM_000518.4|_HBB__mRNA       TGGTCTACCCTTGGACCCAGAGGTTCTTTGAGTCCTTTGGGGATCTGTCC
                                    ********* ****************************************

gi|VK2/E6E7cells|_HBB__mRNA         ACTCCTGATGCTGTTATGGGCAACCCTAAGGTGAAGGCTCATGGCAAGAA
gi|ref|NM_000518.4|_HBB__mRNA       ACTCCTGATGCTGTTATGGGCAACCCTAAGGTGAAGGCTCATGGCAAGAA
                                    **************************************************

gi|VK2/E6E7cells|_HBB__mRNA         AGTGCTCGGTGCCTTTAGTGATGGCCTCTCTCACCTGGACAACCTCAAGG
gi|ref|NM_000518.4|_HBB__mRNA       AGTGCTCGGTGCCTTTAGTGATGGCCTGGCTCACCTGGACAACCTCAAGG
                                    ***************************  *********************

gi|VK2/E6E7cells|_HBB__mRNA         GCACCTTTGCCACACTGAGTGAGCTGCACTGTGACAAGCTGCACGTGGAT
gi|ref|NM_000518.4|_HBB__mRNA       GCACCTTTGCCACACTGAGTGAGCTGCACTGTGACAAGCTGCACGTGGAT
                                    **************************************************

gi|VK2/E6E7cells|_HBB__mRNA         CCTGAGAACTTCAGGCTCCTGGGCAACGTGCTGGTCTGTGTGCTGGCCCA
gi|ref|NM_000518.4|_HBB__mRNA       CCTGAGAACTTCAGGCTCCTGGGCAACGTGCTGGTCTGTGTGCTGGCCCA
                                    **************************************************

gi|VK2/E6E7cells|_HBB__mRNA         TCACTTTGGCAAAGAATTCACCCCACCAGTGCAGGCTGCCTATCAGAAAG
gi|ref|NM_000518.4|_HBB__mRNA       TCACTTTGGCAAAGAATTCACCCCACCAGTGCAGGCTGCCTATCAGAAAG
                                    **************************************************

gi|VK2/E6E7cells|_HBB__mRNA         TGGTGGCTGGTGTGGCTAATGCCCTGGCCCACAAGTATCACTAAAGGCAC
gi|ref|NM_000518.4|_HBB__mRNA       TGGTGGCTGGTGTGGCTAATGCCCTGGCCCACAAGTATCACTAA------
                                    ********************************************

gi|VK2/E6E7cells|_HBB__mRNA         CGAGCACTTTCTTGCCATGAGCCTTCACCTTAGGGTTGC------CCATA
gi|ref|NM_000518.4|_HBB__mRNA       -GCTCGCTTTCTTGCTGTCCAATTTCTATTAAAGGTTCCTTTGTTCCCTA
                                     *  * *********  *     ***   * * **** *      ** **

gi|VK2/E6E7cells|_HBB__mRNA         A---CAGCATCAGGAGTGGACAGATCTTTAAAGGACTCAAAGAACCT---
gi|ref|NM_000518.4|_HBB__mRNA       AGTCCAACTACTAAACTGGGGGATATTATGAAGGGCCTTGAGCATCTGGA
                                    *   ** *  *   * ***       * * **** *    ** * **

gi|VK2/E6E7cells|_HBB__mRNA         CTGGGTCCAAGGGTAGACCACCAGCAGCCTGCCC
gi|ref|NM_000518.4|_HBB__mRNA       TTCTGCCTAATAAAAAACATTTATTTTCATTGC-
                                     *  * * **    * **    *    * *  *
```

```
CLUSTAL 2.1 Multiple Sequence Alignments


Sequence type explicitly set to DNA
Sequence format is Pearson
Sequence 1: gi|VK2/E6E7cells|_HBB__mRNA     535 bp
Sequence 2: gi|ref|NM_000518.4|_HBB__mRNA   626 bp
Start of Pairwise alignments
Aligning...



Sequences (1:2) Aligned. Score: 74.0187
Guide tree file created:   [clustalw.dnd]

There are 1 groups
Start of Multiple Alignment

Aligning...
Group 1: Sequences:   2      Score:8561
Alignment Score 3174

CLUSTAL-Alignment file created  [clustalw.aln]
```

Figure D. Clustal W alignment of sequenced Hb-β PCR product. Hb-β (HBB) sequence is having the accession number, NM_000558.4

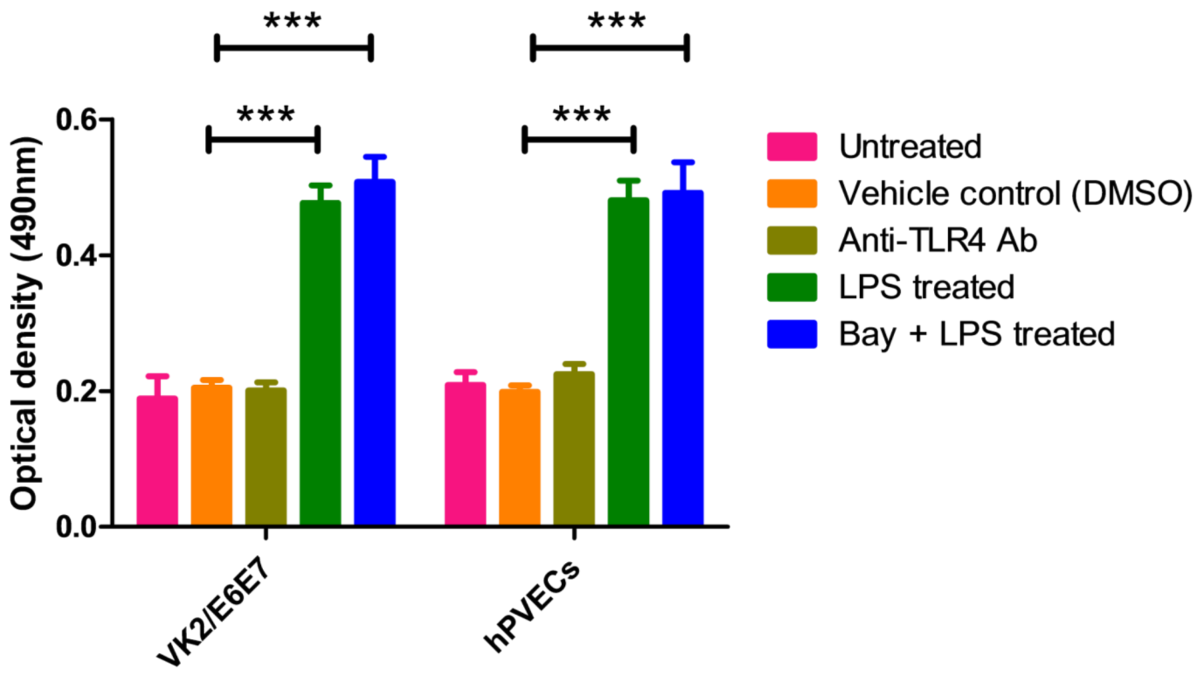

Figure E. TLR4 levels in untreated control, DMSO treated control, LPS stimulated and Bay 11 7082 treated hPVECs and VK2/E6E7 cells analysed by ELISA. Cells were seeded at a density of 10^6/well in a 24-well plate and induced with LPS (10 µg/ml for 6 hrs) or Bay 11-0782 (5µM for 24 hrs) . Expression of TLR4 was up-regulated in LPS-induced cells. Bay 11-7082 treated cells induced with LPS, the levels of TLR4 were up-regulated. In cells treated with anti-TLR4 antibody, the levels of TLR4 were at par with the controls. Values represent the mean ± SD of three experiments performed on different days. All the values are statistically significant (***p<0.001 over the controls).

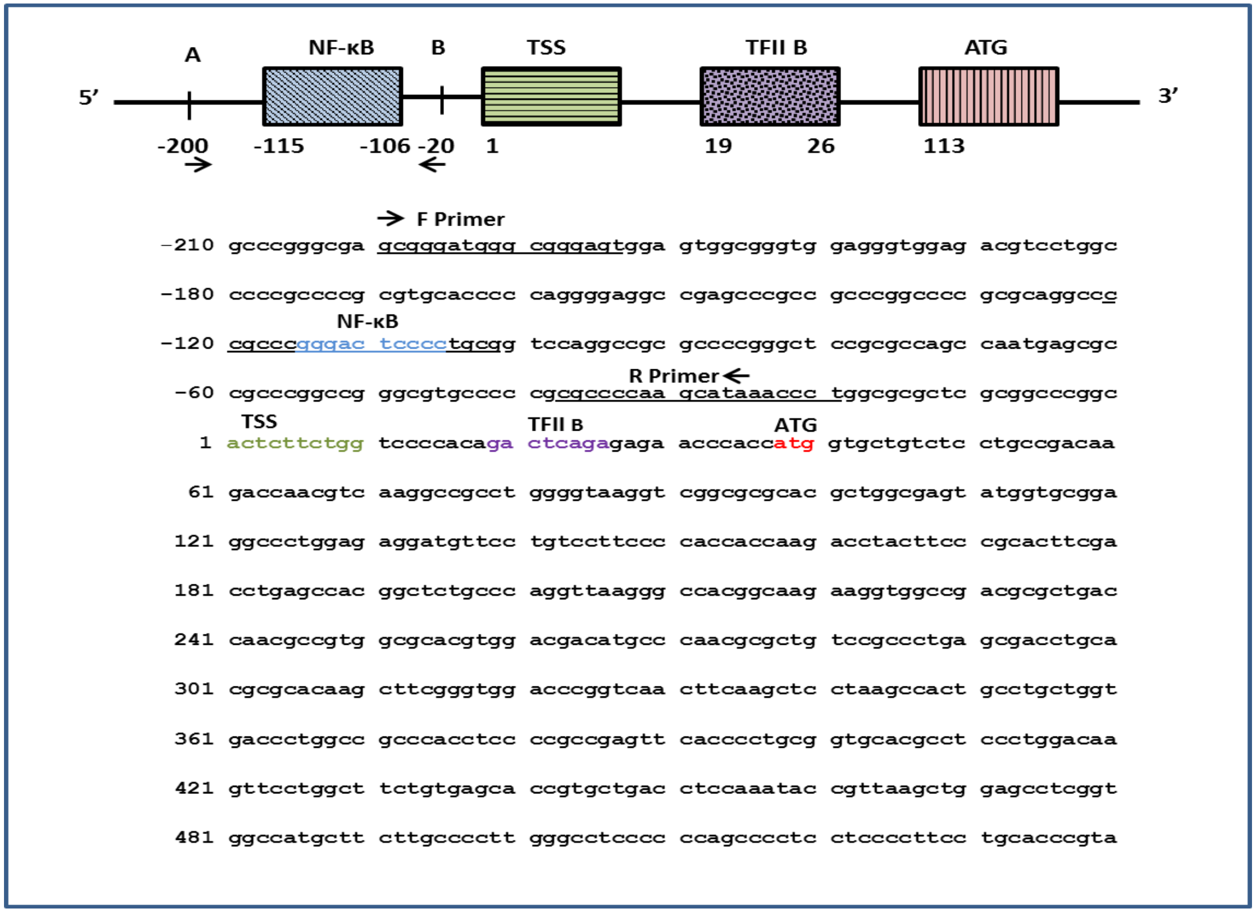

Figure F. Schematic representation of the human Hb-α promoter upstream region (-210 to +540) (A). The -5' (A) and -3' (B) primers used for the Hb-α promoter amplification are shown. TSS, NF-κB, TFIIB and ATG sites are indicated. (TSS: transcription start site, TFIIB: Transcription factor binding site, NF-κB: Nuclear factor kappaB). NF-κB primers used in ChIP assay are indicated (arrows).

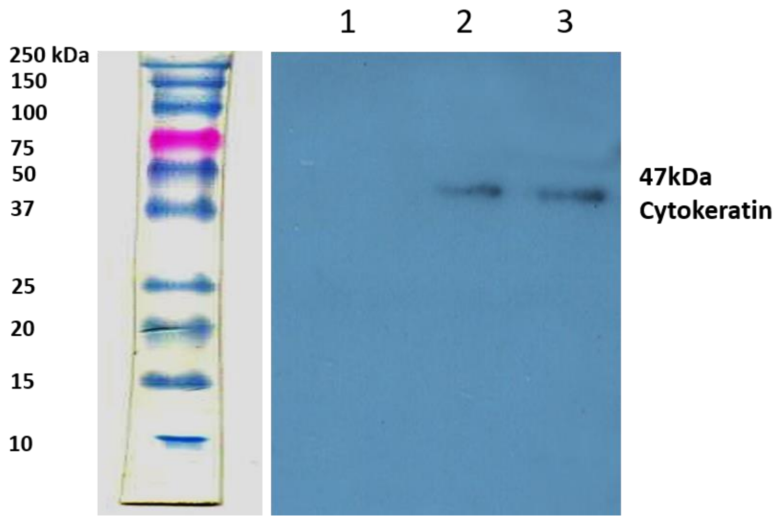

Figure G. Western blot (Uncut Fig-4a)

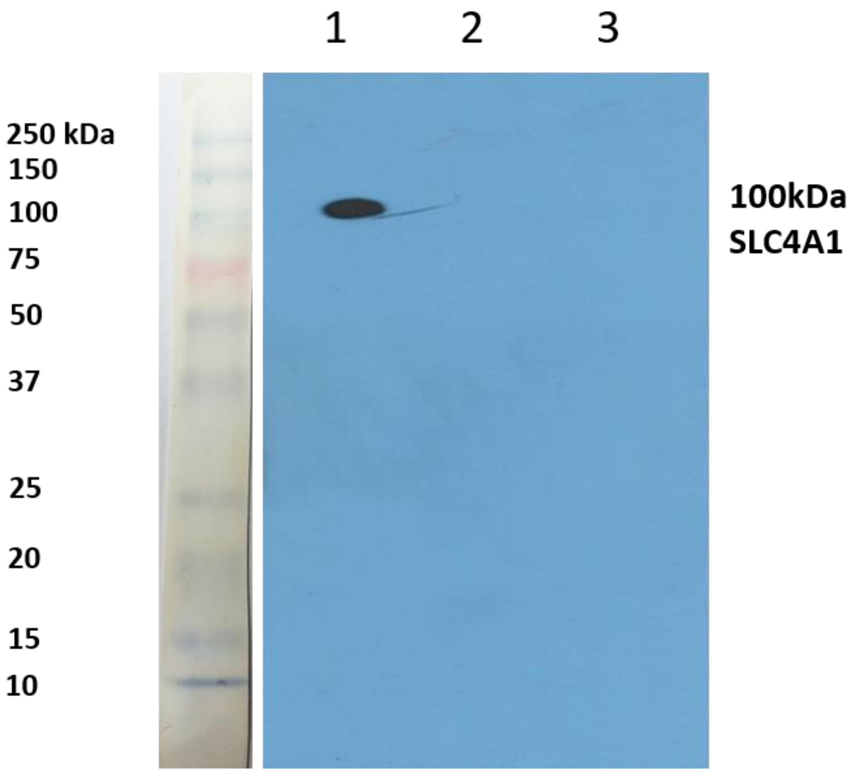

Figure H. Western blot (Uncut Fig4b)

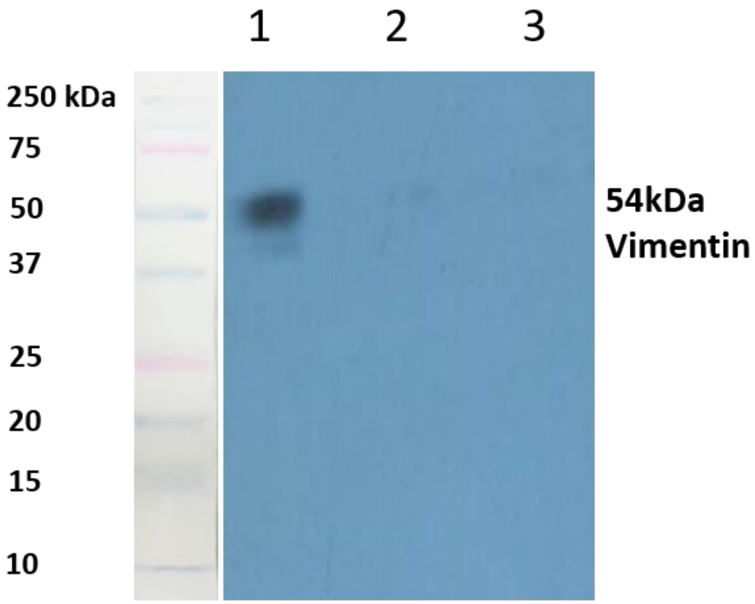

Figure I. Western blot (Uncut Fig4c)

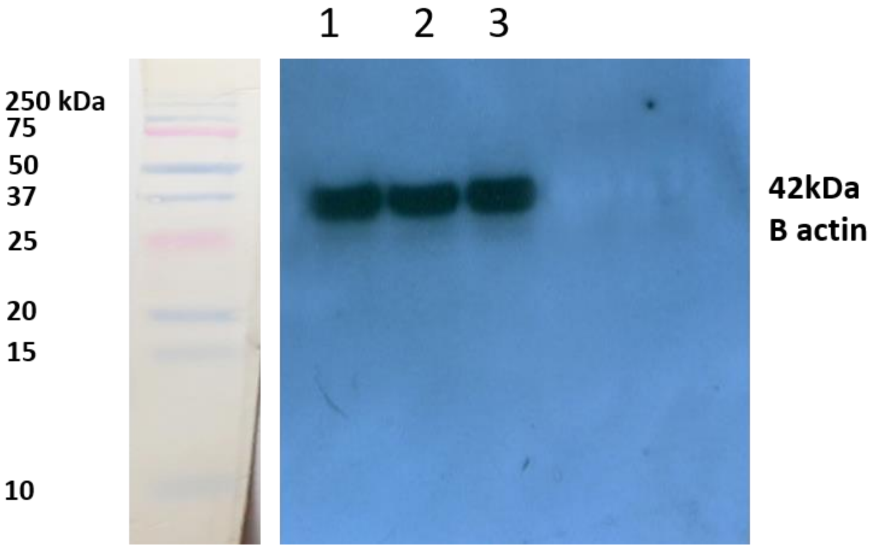

Figure J. Western blot (Uncut Fig4d)

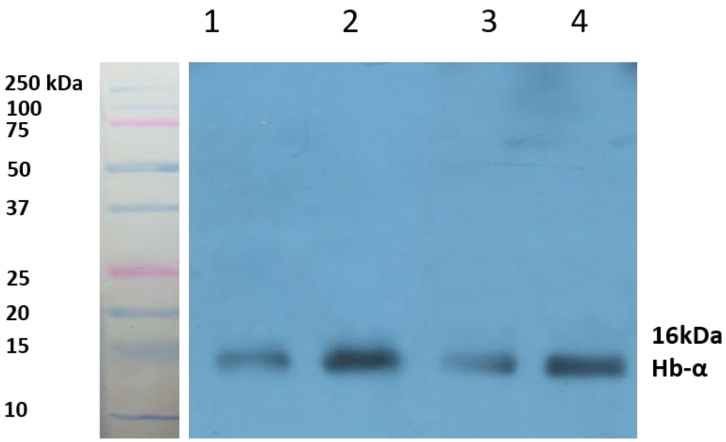

Figure K. Western blot (Uncut Fig7a)

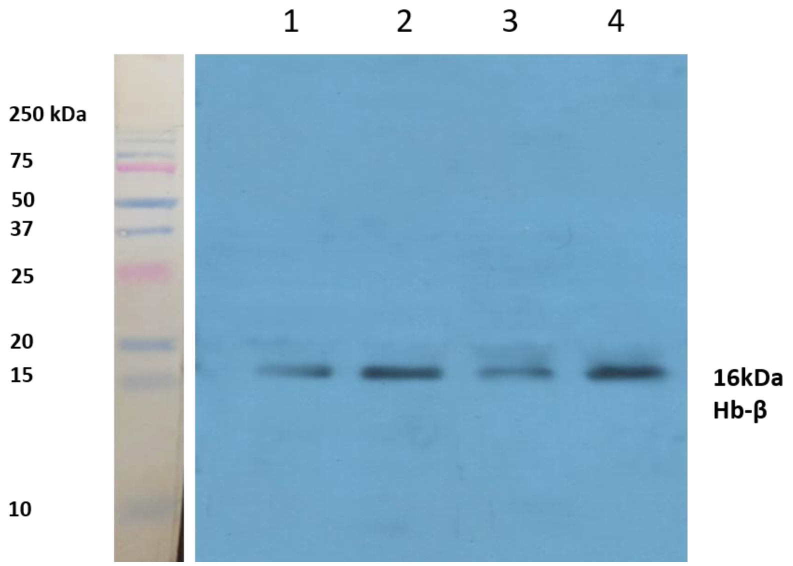

Figure L. Western blot (Uncut Fig7b)

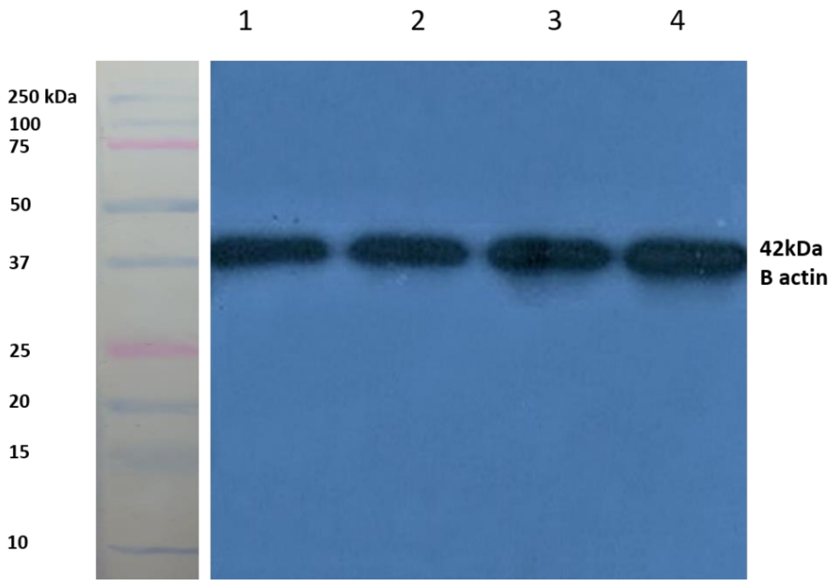

Figure M. Western blot (Uncut Fig7c)

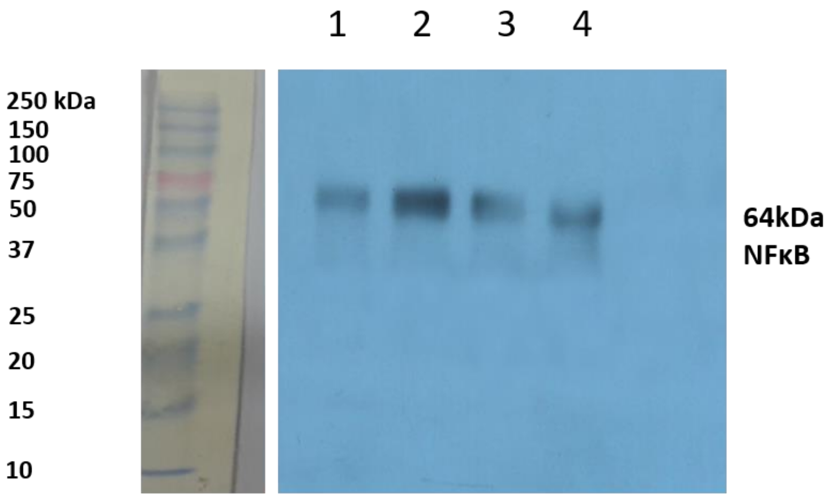

Figure N. Western blot (Uncut Fig-9b, i)

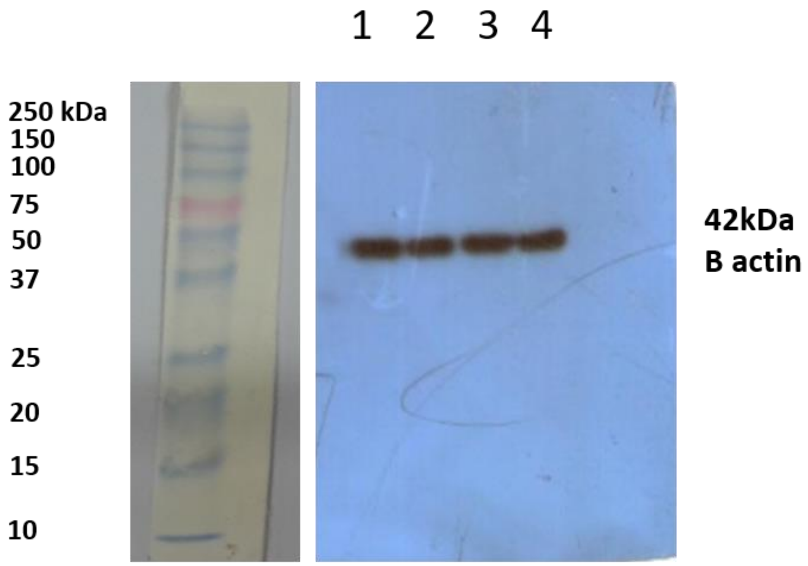

Figure O. Western blot (Uncut Fig9b, ii)

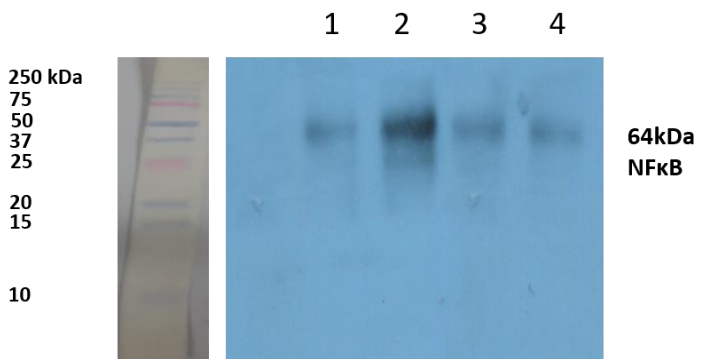

Figure P. Western blot (Uncut Fig9b, iii)

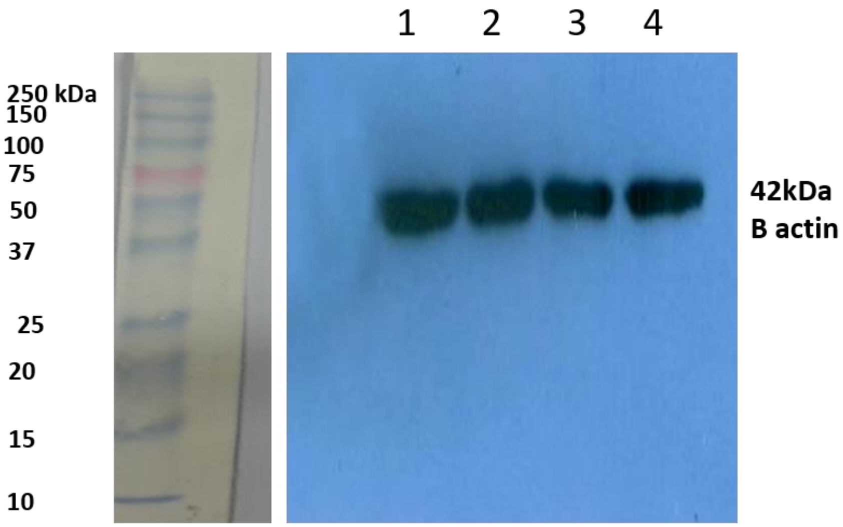

Figure Q. Western blot (Uncut Fig9b, iv)

Table A. Densitometry values of Fig5B.

Parameter	hPVECs (Untreated)							hPVECs (LPS treated)							VK2/E6E7 (Untreated)							VK2/E6E7 (LPS treated)					
Hb-α	0.239	0.229	0.259	0.269	0.301	0.291		0.512	0.0.489	0.506	0.53	0.562	0.586		0.197	0.203	0.195	0.189	0.18	0.173		0.871	0.888	0.813	0.227	0.816	0.823
Hb-β	0.707	0.663	0.745	0.759	0.83	0.716		0.888	0.912	0.967	0.943	1.04	0.944		0.218	0.224	0.217	0.209	0.273	0.265		0.96	0.86	0.947	0.938	0.97	982

Table B. Fold change values of Fig6.

Parameter	hPVECs (Untreated)							hPVECs (LPS treated)							VK2/E6E7 (Untreated)							VK2/E6E7 (LPS treated)					
Hb-α	1	1	1	1	1	1		2.02	2.033	1.955	1.879	2.207	2.202		1	1	1	1	1	1		2.399	2.158	2.36	2.012	2.08	2.396
Hb-β	1	1	1	1	1	1		2.206	2.166	2.201	2.339	2.541	2.171		1	1	1	1	1	1		2.49	2.127	2.319	2.142	2.598	2.31

Table C. Densitometry values of Fig7D.

Parameter	hPVECs (Untreated)							hPVECs (LPS treated)							VK2/E6E7 (Untreated)							VK2/E6E7 (LPS treated)					
Hb-α	0.132	0.148	0.21	0.2	0.24	0.28		0.71	0.82	0.761	0.839	0.81	0.88		0.148	0.132	0.21	0.19	0.25	0.27		0.88	0.68	0.7	0.71	0.89	0.77
Hb-β	0.27	0.168	0.23	0.27	0.33	0.29		0.66	0.68	0.761	0.765	0.8	0.78		0.175	0.185	0.23	0.27	0.34	0.24		0.76	0.78	0.85	0.75	0.87	0.85

Table D. ELISA values of Graph 9A.

Parameter	Control Untreated							LPS treated							BAY treated					
p65 NF-kB (hPVECs)	0.196	0.176	0.193	0.191	0.184	0.225		0.449	0.377	0.431	0.423	0.448	0.434		0.191	0.157	0.186	0.192	0.173	0.219
Total NF-kB (hPVECs)	0.222	251	0.222	0.262	0.259	0.25		0.427	0.436	0.445	0.443	0.453	0.451		0.202	0.233	0.189	0.25	0.227	0.237
p65 NF-kB (VK2/E6E7)	0.194	0.192	0.189	0.213	0.226	0.205		0.433	0.44	0.411	0.478	0.422	0.291		0.143	0,141	0.153	0.147	0.168	0.166
Total NF-kB (VK2/E6E7)	0.202	0.22	0.202	0.245	0.206	0.268		0.46	0.479	0.434	0.461	0.475	0.451		0.189	0.222	0.204	0.226	0.212	0/246

Table E. Densitometry values of Fig9C.

Cells	Untreated							LPS treated							Bay treated							Bay + LPS treated					
hPVECs	0.461	0.385	0.421	0.44	0.45	0.498		0.888	0.846	0.76	0.98	0.936	0.95		0.237	0.236	0.289	0.271	0.33	0.25		0.291	0.249	0.25	0.33	0.23	0.19
VK2/E6E7	0.33	0.27	0.257	0.383	0.269	0.411		0.81	0.83	0.91	0.87	0.88	0.76		0.12	0.1	0.21	0.17	0.137	0.123		0.112	0.116	0.174	0.188	0.137	0.127

Table F. Densitometry values of Fig10B

Cells	Untreated							Vehicle Control (DMSO)							Anti TLR4 Ab							LPS treated					
VK2/E6E7	0.051	0.059	0.055	0.065	0.047	0.037		0.067	0.055	0.051	0.059	0.075	0.067		0.061	0.77	0.071	0.072	0.066	0.09		0.37	0.398	0.371	0.421	0.381	0.399
hPVECs	0.117	0.103	0.116	0.12	0.112	0.13		0.117	0.113	0.102	0.136	0.118	0.128		0.132	0.16	0.154	0.172	0.152	0.15		0.44	0.448	0.44	0.466	0.252	0.472

Table G. ELISA values of Graph 11A.

Time (Hrs)	Control hPVECs							LPS hPVECs							Control VK2/E6E7						LPS VK2/E6E7					
6	212	264	251	235	301	267		333	321	389	413	477	473		222	214	243	257	281	321	381	371	421	447	503	481
12	211	207	250	248	265	313		499	467	570	556	666	620		169	149	267	265	370	376	497	505	572	582	650	658
24	202	242	231	249	302	270		331	355	420	402	483	475		162	174	244	238	304	324	347	341	401	445	479	525

Table H. ELISA values of Graph 11B.

Time (Hrs)	Control hPVECs							LPS hPVECs							Control VK2/E6E7							LPS VK2/E6E7					
6	37	45	43	47	47	51		30	28	38	30	38	40		38	40	43	35	48	50		33	35	39	37	44	40
12	47	39	56	42	54	56		29	27	31	33	37	35		33	39	43	44	47	53		26	30	33	35	30	41
24	51	39	51	47	59	47		29	23	35	27	39	33		37	38	41	47	51	52		24	28	32	30	37	36

Table I. Densitometry values of Fig12B

Parameter	hPVECs (Untreated)							hPVECs (Bay treated)							VK2/E6E7 (Untreated)							VK2/E6E7 (Bay treated)					
Hb-α	0.423	0.401	0.42	0.442	0.451	0.457		0.137	0.151	0.165	0.181	0.206	0.208		0.524	0.526	539	0.523	0.538	0.554		0.223	0.227	0.257	0.249	0.281	0.289
Hb-β	0.411	0.437	0.477	0.431	0.478	0.494		0.152	0.174	0.187	0.177	0.199	0.211		0.443	0.419	0.445	0.439	0.441	0.477		0.231	0.215	0.198	0.202	0.188	0.18

Table J. Densitometry values of Fig13B

Parameter	hPVECs (Untreated)							hPVECs (LPS treated)							VK2/E6E7 (Untreated)							VK2/E6E7 (LPS treated)					
hBD-1	0.44	0.38	0.42	0.44	0.49	0.43		0.93	0.79	0.87	0.95	0.95	0.93		0.31	0.3	0.35	0.33	0.37	0.35		0.81	0.75	0.78	0.82	0.84	0.82
